# Co-Administration of Iron and Bioavailable Curcumin Reduces Levels of Systemic Markers of Inflammation and Oxidative Stress in a Placebo-Controlled Randomised Study

**DOI:** 10.3390/nu14030712

**Published:** 2022-02-08

**Authors:** Helena Tiekou Lorinczova, Gulshanara Begum, Lina Temouri, Derek Renshaw, Mohammed Gulrez Zariwala

**Affiliations:** 1 Centre for Nutraceuticals, School of Life Sciences, University of Westminster, 115 New Cavendish Street, London W1W 6UW, UK; w1505041@my.westminster.ac.uk (H.T.L.); begumru@westminster.ac.uk (G.B.); lina_temouri@hotmail.co.uk (L.T.); 2 Centre for Sport, Exercise and Life Sciences, Institute for Health and Wellbeing, Coventry University, Priory Street, Coventry CV1 5FB, UK; derek.renshaw@coventry.ac.uk

**Keywords:** curcumin, iron, ferrous sulphate, supplementation, inflammation, antioxidant

## Abstract

Ferrous sulphate (FS) is widely used as an iron supplement to treat iron deficiency (ID), but is known to induce inflammation causing gastric side-effects resulting in poor adherence to supplement regimens. Curcumin, a potent antioxidant, has been reported to suppress inflammation via down regulation of NF-κB. The aim of the present double blind, placebo-controlled randomised trial was to assess whether co-administration of FS with a formulated, bioavailable form of curcumin (HydroCurc™) could reduce systemic inflammation and/or gastrointestinal side-effects. This study recruited 155 healthy participants (79 males; 26.42 years ± 0.55 and 76 females; 25.82 years ± 0.54), randomly allocated to one of five different treatment groups: iron and curcumin placebo (FS0_Plac), low dose (18 mg) iron and curcumin placebo (FS18_Plac), low dose iron and curcumin (FS18_Curc), high dose (65 mg) iron and curcumin placebo (FS65_Plac), and high dose iron and curcumin (FS65_Curc). Completed questionnaires and blood samples were collected from all participants at baseline (day 1), mid-point (day 21), and at end-point (day 42). Results showed a significant reduction in IL-6 in the FS65_Curc group (0.06 pg/mL ± 0.02, *p* = 0.0073) between the mid-point and end-point. There was also a significant reduction in mean plasma TNF levels in the FS65_Curc (0.65 pg/mL ± 0.17, *p* = 0.0018), FS65_Plac (0.39 pg/mL ± 0.15, *p* = 0.0363), and FS18_Curc (0.35 pg/mL ± 0.13, *p* = 0.0288) groups from mid-point to end-point. A significant increase was observed in mean plasma TBARS levels (0.10 µM ± 0.04, *p* = 0.0283) in the F18_Plac group from baseline to end-point. There was a significant association with darker stools between FS0_Plac vs. FS65_Plac (*p* = 0.002, Fisher’s exact test) suggesting that high iron dose in the absence of curcumin leads to darker stools. A reduction in inflammation-related markers in response to co-administering supplemental iron alongside formulated curcumin suggests a reduction in systemic inflammation. This supplementation approach may therefore be a more cost effective and convenient alternative to current oral iron-related treatments, with further research to be conducted.

## 1. Introduction

Iron is an essential nutrient that plays a vital role in many biological processes necessary to maintain life [1,2]. Iron deficiency (ID) is the most prevalent nutritional deficiency [3] affecting around a third of the global population [4,5]. Groups who are most at risk of ID are pre-menopausal women [6] and patients with inflammatory bowel disease (IBD) [7,8]. Inadequate dietary intake of iron results in both physical and psychological consequences such as fatigue, adverse pregnancy outcomes, cognitive decline, and reduced mood and quality of life [3,9,10,11,12,13]. Iron supplements such as ferrous sulphate (FeSO_4_) are most commonly used to treat ID, however, these are known to induce associated side-effects such as gastrointestinal (GI) disruption, constipation, diarrhoea, inflammation of the bowel, and black stools [14]. A meta-analysis of randomised controlled trials demonstrated that oral ferrous iron supplementation significantly increased the risk of GI specific side-effects compared to intra-venous ferrous iron or placebo controls and that the side-effects were not related to the dose of oral ferrous sulphate administered [15].

GI side-effects of oral iron supplementation are most likely attributed to two main factors: (i) iron-induced redox cycling in the gut lumen and at mucosal surfaces that induces free radical generation which can promote localised inflammation [16,17,18] and (ii) iron induced effects on the gut micro-biota [19,20,21,22]. Recently, studies on rodents demonstrated that ferrous sulphate supplementation led to increased levels of the hormone hepcidin, as well as the inflammation-related biomarkers interleukin 6 (IL-6) [23,24], tumour necrosis factor (TNF), and thiobarbituric acid reactive substances (TBARS) [24]. Additionally, human clinical studies administering ferrous sulphate supplementation also demonstrated increased circulating levels of IL-6 in pregnant women [25] and haemodialysis patients [26]. Paradoxically, these studies also resulted in significant decreases in total serum iron and serum ferritin concentrations. It has been suggested that these results may be indicative of ferrous sulphate accumulation inside host cells leading to pro-inflammatory conditions [27,28,29]. Serious side-effects of iron supplementation lead to high levels of non-adherence to the supplementation regimen [14,30].

Antioxidants have been demonstrated to counteract the detrimental side-effects of free radicals [31]. Curcumin, a polyphenol derived from turmeric, may exist in keto or enol forms relative to its environment [32]. Curcumin has been reported to negate the deleterious effects of iron-induced reactive oxygen species (ROS) formation, by acting as a free radical scavenger [33,34,35,36] due to the enol form being a hydrogen donor as well as acceptor [37]. Additionally, the enol form of curcumin expresses metal chelation properties in a dose-dependent manner by binding ferric iron (Fe^3+^) [38]. Previous research in animals has shown that whole turmeric or curcumin is strongly associated with a negative impact on iron absorption [39,40,41]. Whole turmeric is known to have similar effects in humans, with 20–90% reduction in iron absorption [42]. Although the mechanisms of iron and curcumin interaction and their effect on the human physiology are not yet well understood, a recent study showed that a formulated bioavailable form of curcumin had no negative impact on acute iron absorption [43]. This formulation (HydroCurc™) consisted of 80% curcumin, 17% demethoxycurcumin (DMC), and 3% bisdemethoxycurcumin (BDMC), ~85% total curcuminoids, entrapped in a patented delivery system (LipiSperse^®^). The delivery system comprises of a proprietary mixture of surfactants, polar lipids, and solvents that can complex lipophilic active ingredients with otherwise poor solubility to be rendered cold water dispersible, thereby increasing their pharmacokinetic profile and overall oral bioavailability. In case of curcumin, the components of LipiSperse^®^ have been shown to embed into its lipophilic crystal structure thus increasing wettability, enhancing dispersibility, and significantly increasing overall plasma concentrations and hence bioavailability in humans [44].

Furthermore, curcumin also counters inflammation by additional mechanisms including down-regulation of NF-κB (nuclear factor kappa-light-chain-enhancer of activated B cells); a transcription factor that is pivotal to the production of pro-inflammatory cytokines [45]. NF-κB acts as a master regulator of inflammation, by controlling the expression of several hundred gene products linked with inflammation and cellular activities including survival, proliferation, angiogenesis, and metastasis [46]. Therefore, finding compounds that reduce NF-κB activity may be of wide benefit. Curcumin is well tolerated in humans with several clinical trials suggest that 8–12 g/day can be administered with no adverse effects reported [47]. 

The aim of the current research was to assess whether co-administration of ferrous sulphate with a formulated, bioavailable form of curcumin (HydroCurc™) could reduce systemic inflammation, gastrointestinal side-effects, and/or reduce fatigue severity associated with iron supplementation in humans. To the authors’ knowledge, a randomised controlled trial of this kind has not been previously reported.

## 2. Materials and Methods

### 2.1. Study Design

Primary outcome measures were systemic inflammation-related markers including IL-1, 6, and 10, TNF, and TBARS. All other measures including subjective markers and questionnaire data was a secondary outcome measure. This study applied a double blind, placebo-controlled randomised trial design. Study Randomizer software [48] was used to carry out blocked, gender-balanced randomisation which is compliant with Health Insurance Portability, General Data Protection Regulation (GDPR), and Accountability Act (HIPAA). G*Power 3.1.9.2 software statistical analysis software (Kiel, Germany) [49,50] aided the calculation of the sample size based on moderate effect size, calculating with a rate of 10% sample loss.

### 2.2. Ethics Approval and Study Registration

The study protocol was approved by the Faculty of Science and Technology Ethics Committee, University of Westminster (application identification ETH1718-0907). The study is registered with ClinicalTrials.gov (accessed on 10 January 2022) (NCT04465851).

### 2.3. Inclusion and Exclusion Criteria for Participants

Inclusion criteria were for participants to be generally healthy, aged between 18 and 40 years, with ferritin levels within the normal range for the United Kingdom (UK; 15–300 µg/L for men and 15–200 µg/L for women) as defined by Dooley and Worwood, and Fitzsimons and colleagues [51,52]. 

Exclusion criteria included individuals with deficient haemoglobin (HGB) levels (<130 g/L for men and <120 g/L for women), any diagnosis of medical conditions or comorbidities (currently trying to conceive, pregnant, lactating, experiencing any chronic menstrual disorders, or reported undergoing any menopausal changes). Participants with any issues related to ingesting oral supplementation, taking any supplementation or medication, with alcohol consumption exceeding 21 units/week, chronic GI symptoms, eating disorders, psychological conditions, or any hypo/hypertensive blood pressure (BP) measurements [53,54] were also excluded from the study. 

### 2.4. Safety Screening

A 60 min medical history interview was held at the University of Westminster, London, UK, W1W 6UW. Potential participants were interviewed for study suitability using open-ended questions to screen for any health conditions that may prevent participation. The interview was supported by the use of the General Symptoms Questionnaire (GSQ-65) as developed by Hyland (2017). The GSQ-65 was validated for use across different diagnostic categories using frequency and factor analysis [55]. 

Additionally, a fasting (12 h, overnight) blood sample was collected for haemoglobin and ferritin measurements as part of the inclusion criteria highlighted in Section 2.3. BP screening was carried out using the Omron M6 Comfort blood pressure monitor (Omron, Hoofddorp, The Netherlands). Participants were excluded from the study if anomalous results or findings were established. 

### 2.5. Treatment Groups

**Figure 1:**
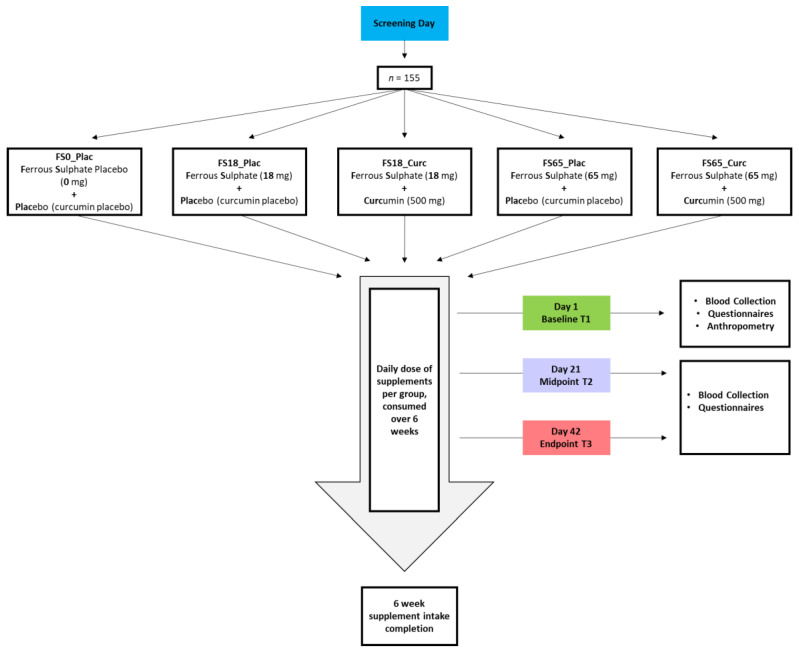
Overview of study. Participants meeting study inclusion criteria during screening were randomly assigned to one of the five treatment groups. At baseline (day 1) anthropometric measurements, blood samples, and questionnaires were obtained from the participants. At mid-point (day 21) and end-point (day 42), blood samples and questionnaires were collected from the study volunteers.

Participants were randomly allocated to one of five different treatment groups for a duration of 6 weeks. The five different treatment groups were ferrous sulphate placebo + curcumin placebo (FS0_Plac), ferrous sulphate—18 mg elemental iron + placebo (FS18_Plac), ferrous sulphate—18 mg elemental iron + 500 mg curcumin (FS18_Curc), ferrous sulphate—65 mg elemental iron + placebo (FS65_Plac), and ferrous sulphate—65 mg elemental iron + 500 mg curcumin (FS65_Curc) (Figure 1).

### 2.6. Supplements

Depending on the supplement group allocation (Figure 1), participants were provided with either a high or low dose of ferrous sulphate supplement, co-administered with a dose of curcumin or equivalent placebo(s). Details of the supplements have been previously described in detail in Tiekou Lorinczova and colleagues [56]. Briefly, curcumin supplements comprised of 500 mg/day of formulated curcumin (HydroCurc™, Pharmako Biotechnologies Pty Ltd., Sydney, Australia). The supplements were presented in white, screw lid bottles, labelled with the group code. Instructions for consumption were disclosed to participants; one ferrous sulphate and one curcumin supplement to be taken daily with water, at least 1 h before or 2 h after food consumption, at separate time intervals. 

### 2.7. Physical Examination

Participants’ baseline anthropometric measurements were collected on day 1 of the study by trained staff. Seca 287 ultrasonic stadiometer (Seca GmbH & Co. KG, Hamburg, Germany) was used to measure height (m). Weight (kg) and body mass index (BMI; kg/m^2^) were measured using the Seca 515 medical Body Composition Analyser (Seca GmbH & Co. KG, Hamburg, Germany). Prior to conducting measurements, participants were instructed to empty pockets, remove any excessive clothing and any jewellery, and void bladder of urine prior to all measurements. 

### 2.8. Blood Sample Collection and Processing 

Blood samples were collected following an overnight fast (12 h fast) for each participant. Approximately 10 mL of venous blood samples were drawn from the antecubital fossa via venepuncture (using a 21 G needle) by certified phlebotomists at baseline, mid-point (day 21), and end-point (day 42) visits. Blood was collected using Becton Dickinson (BD) Vacutainer^®^ serum-separating tubes (SST) (BD, Oxford, UK). Blood samples in the SST were kept at room temperature for 45 min (to coagulate) and then centrifuged (Hettich 340 r, Hettich GmbH & Co. KG, Tuttlingen, Germany) for 10 min at 3857× *g*. Aliquots of 1.5 mL serum supernatant were stored at −80 °C post-centrifugation, prior to analysis. 

### 2.9. Analysis of Serum Ferritin Concentration and C-Reactive Protein (CRP)

Defrosted serum ferritin and plasma CRP samples were analysed using a Horiba ABX Pentra 400 (Horiba Ltd., Kyoto, Japan) multiparametric medical benchtop chemistry analyser and the Horiba ABX Pentra CRP CP High Sensitivity and ABX Ferritin 2 CP reagents, compliant with the National Committee for Clinical Laboratory Standards (NCCLS) [57]. Ferritin and CRP levels were determined by latex-enhanced immunoturbidimetric assay [58] in accordance with the manufacturer’s processes. 

### 2.10. Analysis of Serum Iron Profile

Serum iron (Fe), total iron binding capacity (TIBC), transferrin saturation (TS), and unsaturated iron-binding capacity (UIBC) were assessed by a commercial laboratory (Health Services Laboratories; HSL Analytics LLP, London, UK), Project No. P197, using the relevant standards and controls for each analyte). Serum Fe and UIBC were measured by direct colorimetric method and subsequently, serum TIBC and TS were calculated using the equations from Elsayed et al. [59]:

TIBC = Fe + UIBC

TS = Fe/(Fe +UIBC)×100

### 2.11. Analysis of Whole Blood Haemoglobin (HGB) Concentration

Normal haemoglobin HGB levels range from 130–175 g/L for men and 120–155 g/L for women. The Sysmex XP-300 (Sysmex Corporation, Kobe, Japan) automated haematology analyser was used to conduct HGB analysis. The Sysmex XP-300 is compliant with International Standards Organisation (ISO)/International Electrotechnical Commission (IEC) 17043:2010, via the Sysmex Network Communication Service. A total of 50 μL of each sample was aspirated by the Sysmex XP-300. A non-cyanide haemoglobin detection method [60] was used for HGB analysis. Calibration of the XP-300 was performed by Sysmex Corporation according to the manufacturer’s specifications; quality was assured using Sysmex internal quality control. 

### 2.12. Analysis of Interleukin 6 (IL-6), Interleukin 10 (IL-10), Interleukin 1 Beta (IL-1β), and Tumour Necrosis Factor (TNF) 

Plasma samples were assayed using a built-to-order analyte customisable 4 plex cytokine assay. The R&D systems Human High Sensitivity Cytokine plasma specific, multianalyte kit (IL-6, IL-10, IL-1β, and TNF) (R&D Systems, Minneapolis, MN, USA) were used for sample analysis. The assays were performed according to the manufacturer’s instructions. The Luminex Magpix^®^ xPONENT software and plate reader instrument (Luminex Technologies, Austin, TX, USA) were used to perform multianalyte profiling of circulating concentrations of inflammation-related markers. Standard curves were generated for each cytokine and the mean fluorescence intensity (MFI) of each cytokine in each well was corelated to determine concentrations. 

### 2.13. Analysis of Thiobarbituric Acid Reactive Substances (TBARS)

TBARS parameter assay kit (R&D Systems, Minneapolis, MA, USA) was used to determine Serum TBARS. The manufacturer’s instructions were followed to perform the assay; a microplate reader (SPECTROstar^®^ Nano, BMG Labtech GmbH, Ortenberg, Germany) was used to read the plates at 530 nm absorbance. 

### 2.14. Fatigue Severity Scale (FSS)

All study participants were instructed to complete the FSS daily, via the SurveyMonkey^®^ online survey and questionnaire platform (Momentive Inc., San Mateo, CA, USA). The FSS is a 9 question, 7-point Likert scale (1 = strongly disagree and 7 = strongly agree) self-assessed questionnaire. The total score of all answers indicates the level of fatigue (63 = maximum fatigue). Krupp and colleagues (1989) previously stated that a total score above 36 indicates fatigue [61]. The results generated from this tool have also been validated by Lerdal and colleagues (2005) in a healthy Western population [62]. 

### 2.15. Fatigue Visual Analogue Scale (F-VAS)

The FSS is often combined with a simple one-point F-VAS to facilitate expression of one’s perceptions and is considered to be sensitive to change [63,64,65]. The F-VAS was presented to the participants as a one-point linear horizontal line measuring 100 mm. They were instructed to apply a vertical mark closest to where they were feeling along the scale (at the time of measurement). The furthest point to the left (a score of 0) indicates extreme fatigue whereas the furthest point right along the line (a score of 100) indicates normal levels of energy without fatigue. In this study, F-VAS (in addition to FSS) was completed only on day 1, 21, and 42.

### 2.16. Questionnaire to Assess GI Symptoms after Oral Ferrous Iron Supplementation

A modified version of the GI questionnaire developed and validated by Pereira et al. (2014) was used for all study participants. The questionnaire consisted of several sections with particular emphasis on questions that discriminate for oral ferrous iron intake. Three questions/outcomes (heartburn, abdominal pain, and presence of black stools) were noted as being discriminatory for adverse events between oral ferrous iron intake compared to placebo by Pereira and colleagues [66]. Due to the healthy nature of the population in the current study, an additional question was added to the existing questionnaire, related to ‘darkened stools’ in addition to ‘black’ stools as a side-effect of the treatment. Participants were asked to complete the questionnaire daily throughout the study period via the SurveyMonkey^®^ online survey and questionnaire platform. 

### 2.17. Study Compliance and Adverse Reactions 

At mid-point (day 21) and end-point (day 42) of the study period, participant adherence to protocol (including supplementation) and adverse effects were monitored. Any nonadherence to study protocol was noted and assessed. Daily supplementation adherence of the active or placebo capsules was ≥80% at all times. Data from all participants who successfully completed the study period were included in the data analysis (Figure S1).

### 2.18. Statistical Analysis 

Values are expressed as mean ± Standard Error of Mean (SEM). Shapiro–Wilk (S-W) tests were applied to check all parameters for normality. Given the repeated measures design of this study, results were statistically analysed using a two-way, repeated measures analysis of variance (ANOVA) or mixed effects model (where missing values were present). A wide body of evidence in literature [67,68,69] indicates that ANOVAs are generally robust to violations of normality (where present). In addition to the overall analysis, the authors applied subgroup analysis based on previous work [70,71,72] suggesting that a serum ferritin cut-off of 30 ng/mL is indicative of insufficient iron stores. This 30 ng/mL cut off point appears to have greater sensitivity than the 15 ng/mL cut-off recommended by the World Health Organisation [73]. Moreover, serum ferritin below 50 ng/mL has also been suggested to indicate deficient or reduced bone marrow iron stores or latent iron deficiency [72,74]. Accordingly, treatment sub-group data analysis is presented (where relevant), using these cut off points of 30 ng/mL or 50 ng/mL. To assess the differences between and within treatment groups, post-hoc tests (Sidak’s and Tukey’s) were carried out with multiplicity adjusted *p* values reported for each comparison (PRISM software package, Version 8, Graphpad Software Inc., San Diego, CA, USA). Categorical data from questionnaire responses were analysed in terms of association between supplementation and reported side-effects were analysed via application of the Fischer’s exact test (planned comparisons, with Bonferroni correction) using Statistical Package for the Social Sciences (SPSS, version 22, IBM, Armonk, NY, USA). Statistical significance was set at *p* < 0.05 (unless otherwise stated).

## 3. Results

### 3.1. Participant Characteristics

A total of 155 participants (79 males and 76 females) were enrolled in the present double blind, randomised controlled trial. There were 154 participants included in the data analysis, after the exclusion of participant number 105, due to high body mass index (BMI ≥ 40 kg/m^2^) that qualifies as category 3 obesity and was also identified as 3*IQR or an extreme outlier in SPSS. Daily supplementation adherence of the active or placebo capsules was ≥80% at all times. Overall, the mean age of the included 154 participants was 26.12 years (±0.39). In addition, mean height was 1.71 m (±0.01), and mean weight was 68.93 kg (±1.13). Mean BMI for all treatment groups was within the normal range (18.5 to 24.9). The participant characteristics for five treatment groups are presented in Table 1. Ethnicity data (%) for study participants were Caucasian 64.3, Asian 22.1, African 3.2, Central or South American 2.6, other 7.8. Comprehensive study results for all ferritin cut-off points and whole cohort analysis for all quantitative markers are shown in Supplementary Table S1.

### 3.2. Inflammatory Biomarkers

#### 3.2.1. Effect of Supplementation on Plasma CRP Levels

Intra-group analysis did not show any statistically significant differences for baseline levels when comparing the five supplement groups. There was no statistically significant change observed in plasma CRP intra- or inter-group comparisons as a result of iron plus curcumin administration over the duration of 42 days, when analysing whole groups. In addition, there were no statistically significant changes observed in plasma CRP levels with sub-group analysis using baseline ferritin levels of 30 ng/mL or 50 ng/mL.

#### 3.2.2. Effect of Supplementation on Plasma IL-6 Levels

At baseline, no significant differences were observed in mean plasma IL-6 levels across the five supplement groups. There was no statistically significant variation in mean plasma IL-6 levels within or between the five supplement groups over the course of the supplementation period, with whole group analysis.

**Figure 2:**
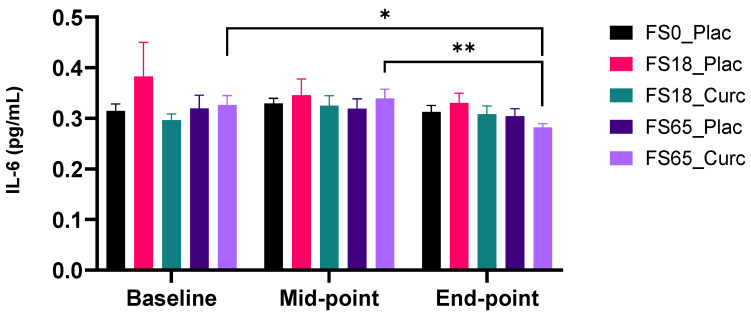
Effect of supplementation on plasma IL-6 levels for participants with ferritin ≥ 30 ng/mL at baseline (*n* = 17–22 per group). Plasma IL-6 was collected and analysed at baseline (day 1), midpoint (day 21), and end-point (day 42). Results are presented as mean ± SEM IL-6 levels (pg/mL). * represents significance values when comparing each condition and time points within the same condition. (* *p* < 0.05, ** *p* < 0.01).

When participants were sub-grouped according to low serum ferritin (<30 ng/mL) and normal serum ferritin (≥30 ng/mL) baseline levels, there were no significant change in mean plasma IL-6 levels over time in any of the supplement groups. There were no significant changes in IL-6 levels with either a low of high dose of ferrous sulphate alone. In the FS65_Curc group (Figure 2), there was a significant reduction in IL-6 (0.06 pg/mL ± 0.02, *p* = 0.0073) between the mid-point and end-point in participants who had ≥30 ng/mL serum ferritin at baseline. Furthermore, in the FS65_Curc group (sub-group with ≥30 ng/mL baseline serum ferritin) there was a significant decrease between baseline to end-point mean plasma IL-6 levels (0.04 pg/mL ± 0.02, *p* = 0.0479).

When participants were sub-grouped according to low and normal serum ferritin baseline levels using <50 ng/mL and ≥50 ng/mL as cut-off points, there were no significant change observed over time in mean plasma IL-6 levels, between the five supplement groups. In the ≥50 ng/mL serum ferritin sub-group (Table S1), there was a significant reduction in plasma IL-6 level response to supplementation in the FS65_Curc group, between the mid-point and end-point (0.04 pg/mL ± 0.01, *p* = 0.0255).

#### 3.2.3. Effect of Supplementation on Plasma TNF

**Figure 3:**
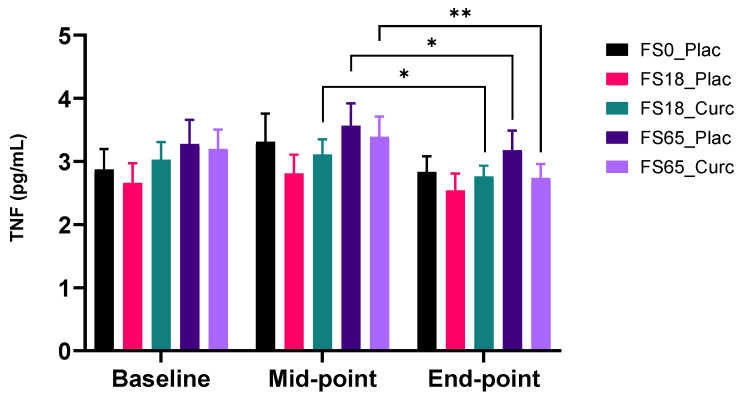
Effect of supplementation on plasma TNF levels (*n* = 29–31 per group). Plasma TNF was collected and analysed at baseline (day 1), mid-point (day 21), and end-point (day 42). Results are presented as mean ± SEM TNF levels (pg/mL). * represents significance values when comparing each condition and time points within the same condition. (* *p* < 0.05, ** *p* < 0.01).

There was no effect of low iron alone (FS18_Plac) on circulating TNF levels at any time point. There was however a significant reduction in TNF levels when comparing mean values in the mid-point to the end-point in the corresponding higher dose iron (FS65_Plac) (0.39 pg/mL ± 0.15, *p* = 0.0363,). In the higher dose iron and curcumin group (F65_Curc), there was a highly significant reduction in plasma TNF levels (0.65 pg/mL ± 0.17, *p* = 0.0018) when comparing mid-point mean levels to end-point mean levels (Figure 3). Furthermore, there was a significant reduction (0.35 pg/mL ± 0.13, *p* = 0.0288) when comparing mid-point to end-point mean levels, in the low dose iron and curcumin group (FS18_Curc) (Figure 3). 

When participants were sub-grouped according to serum ferritin < 30 ng/mL and ≥30 ng/mL baseline levels, there were no significant changes observed in mean plasma TNF levels in the five supplement groups in the <30 ng/mL sub-group. In the ≥30 ng/mL serum ferritin sub-group, there was a significant reduction in plasma TNF levels in FS65_Curc group between the mid-point and end-point (0.69 pg/mL ± 0.23, *p* = 0.0205) (Table S1).

Participants were also sub-grouped according to low serum ferritin (<50 ng/mL) and normal serum ferritin (≥50 ng/mL) levels at baseline. In the group <50 ng/mL serum ferritin, mean levels of TNF showed a decrease in plasma TNF between the mid-point and end-point in the FS65_Curc group (0.73 pg/mL ± 0.19, *p* = 0.0064) (Table S1). 

In the serum ferritin ≥50 ng/mL sub-group, there was no significant change in plasma TNF levels in any of the supplement groups (Table S1).

#### 3.2.4. Effect of Supplementation on Plasma IL-1β

There was no significant intra- or inter-group change observed in mean plasma IL-1β levels in the five supplementation groups when analysing whole groups. There was no significant change in plasma IL-1β levels (inter or intra) in the five supplementation groups when participants were sub-grouped according to low serum ferritin (<30 ng/mL/<50 ng/mL) and normal serum ferritin (≥30 ng/mL/≥50 ng/mL) levels at baseline. For the full data set, see Supplementary Table S1.

#### 3.2.5. Effect of Supplementation on Plasma IL-10 Levels

There was no significant intra- or inter-group change in mean plasma IL-10 levels observed with whole group analysis in the five supplementation groups. There was no significant change in plasma IL-10 levels (inter or intra) in the five supplementation groups when participants were sub-grouped according to low serum ferritin (<30 ng/mL/<50 ng/mL) and normal serum ferritin (≥30 ng/mL/≥50 ng/mL) levels at baseline. For the full data set, see Supplementary Table S1. 

### 3.3. Oxidative Stress Marker

#### Effect of Supplementation on Serum (TBARS) Levels

**Figure 4:**
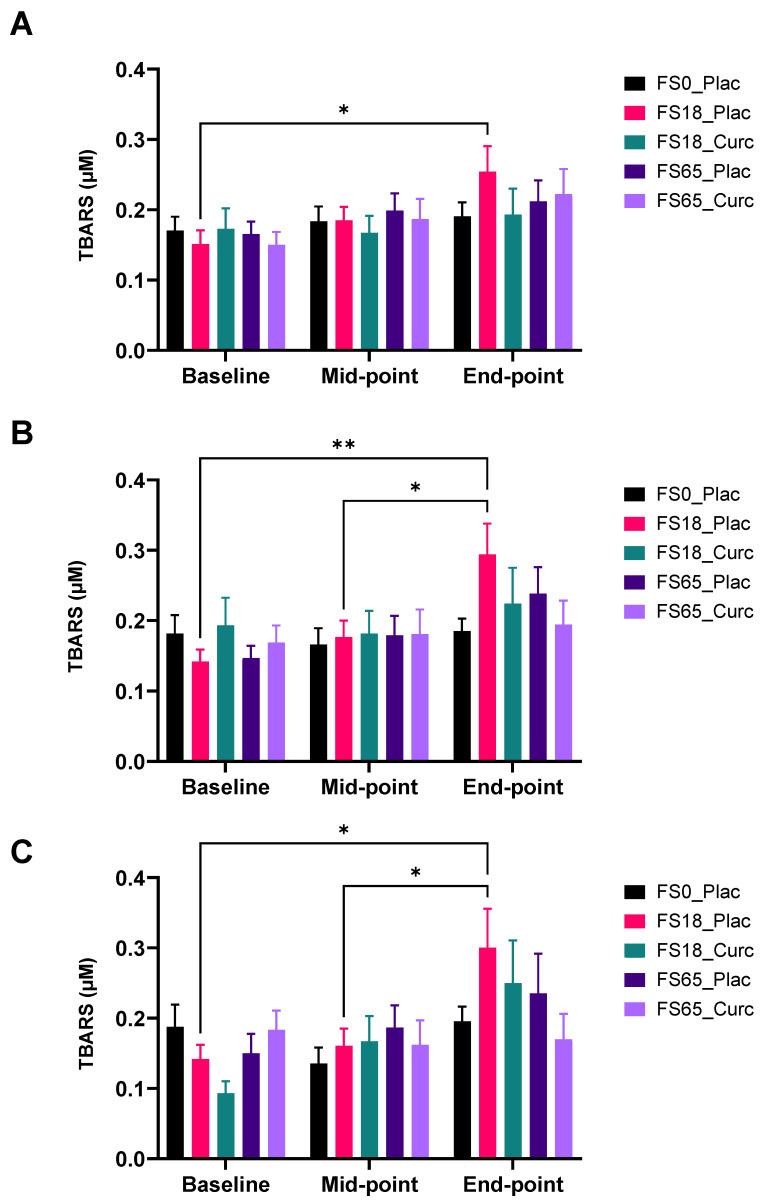
Effect of supplementation on plasma TBARS levels. Plasma TBARS was analysed at baseline (day 1), mid-point (day 21), and end-point (day 42). Results are presented as mean ± SEM TBARS levels (µM) in (**A**) whole group analysis (*n* = 28–30/group), (**B**) participants with ferritin ≥30 ng/mL at baseline (*n* = 17–22/group), and (**C**) participants with ferritin ≥ 50 ng/mL at baseline (*n* = 11–16/group). * represents significance values when comparing each condition and time points within the same condition (* *p* < 0.05, ** *p* < 0.01).

TBARS levels were assessed in all five supplement groups over the study duration. Statistical analysis did not show any significant difference at baseline when comparing between the five supplement groups. In the lower dose iron/placebo group (F18_Plac), there was a highly significant increase in plasma TBARS levels (0.10 µM ± 0.04, *p* = 0.0283), when comparing baseline to end-point mean levels (Figure 4A). 

When participants were sub-grouped according to baseline serum ferritin levels, <30 ng/mL and ≥30 ng/mL, no significant changes were observed in mean plasma TBARS levels among those with low ferritin (<30 ng/mL) at baseline. In the ≥30 ng/mL serum ferritin sub-group, there was a significant increase in plasma TBARS levels in FS18_Plac group between baseline and end-point (0.15 µM ± 0.05, *p* = 0.0079) and mid-point and end-point (0.12 µM ± 0.04, *p* = 0.0217) (Figure 4B).

Furthermore, when participants were sub-grouped according to baseline serum ferritin levels, <50 ng/m and ≥50 ng/mL; mean levels of TBARS showed an increase between mid-point and end-point, among the <50 ng/mL sub-group in the FS18_Plac group (0.10 µM ± 0.04, *p* = 0.0283) (Table S1).

For those within the ≥50 ng/mL sub-group, mean levels of TBARS showed a significant increase in the low iron/curcumin (FS18_Curc) treatment group between baseline and end-point (0.16 µM ± 0.06, *p* = 0.0475) and mid-point and end-point (0.14 µM ± 0.05, *p* = 0.0243) (Figure 4C).

### 3.4. Iron Status Markers

#### 3.4.1. Effect of Supplementation on Serum Ferritin Levels

All five groups were assessed on serum ferritin levels over the study duration. Statistical analysis did not show any significant difference at baseline or between groups at any time point when comparing the five supplement groups. There appeared to be a trend for increased serum ferritin in the FS65_Plac, FS65_Curc, and FS18_Curc groups at both the mid-point and end-point. This trend was also evident when comparing baseline to mid-point mean levels. 

There was a significant mean increase in serum ferritin within the FS65_Plac group (9.61 ng/mL ± 2.42, *p* = 0.0012). Furthermore, there was a statistically significant mean increase from baseline to end-point, within the FS65_Plac group (12.47 ng/mL ± 3.83, *p* = 0.0078). Similarly, in the active comparator group (FS65_Curc), there was a trend for increased mean serum ferritin, albeit non-significant (9.77 ng/mL ± 4.08, *p* = 0.0589) from baseline to end-point (Table S1).

**Figure 5:**
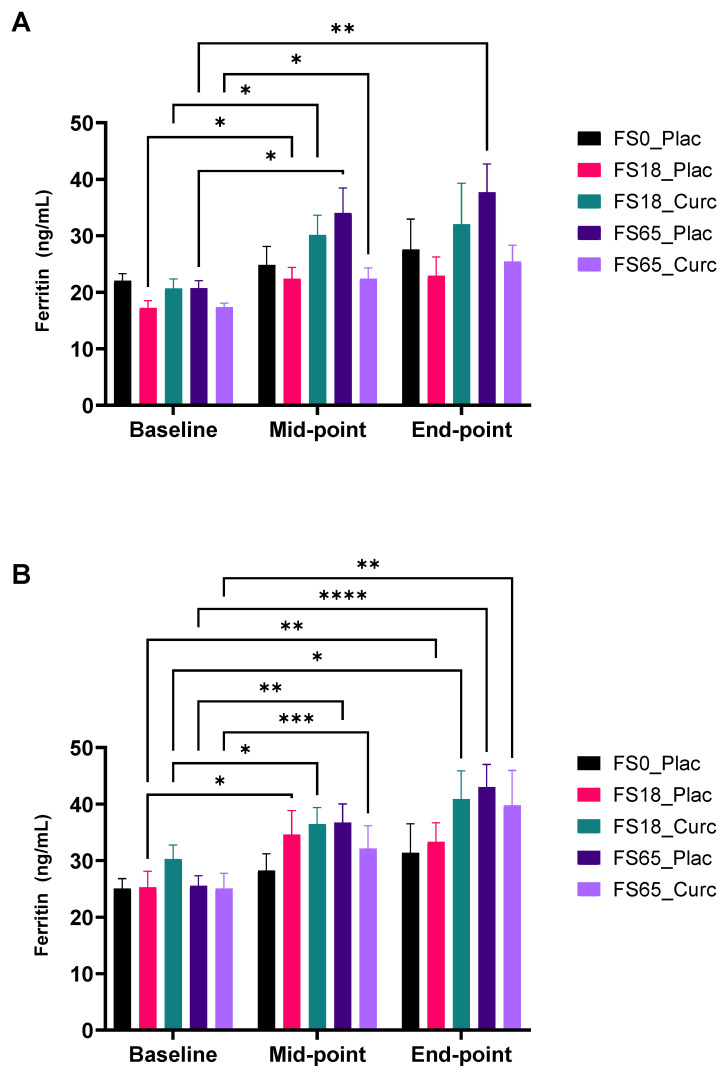
Effect of supplementation on serum ferritin levels. Serum ferritin was collected and analysed at baseline (day 1), mid-point (day 21), and end-point (day 42). Results are presented as mean ± SEM ferritin levels (ng/mL) in (**A**) participants with ferritin < 30 ng/mL at baseline (*n* = 7–13 per group) and (**B**) participants with ferritin < 50 ng/mL at baseline (*n* = 14–21/group). * represents significance values when comparing each condition and time points within the same condition (* *p* < 0.05, ** *p* < 0.01, *** *p* < 0.001, **** *p* < 0.0001).

With respect to subgroup analysis, there was a significant (9.49 ng/mL ± 3.31, *p* = 0.0407) increase between baseline and mid-point, in response to supplementation in FS18_Curc, for those with serum ferritin < 30 ng/mL at baseline (Figure 5A). In the corresponding placebo group (FS18_Plac), there was also a significant (5.18 ng/mL ± 1.31, *p* = 0.0132) increase in serum ferritin, between the baseline and mid-point. In the higher dose iron and curcumin (FS65_Curc) group, there was also a significant (5.04 ng/mL ± 1.70, *p* = 0.0430) increase in serum ferritin levels, between the baseline and mid-point. Similarly, the placebo group (FS65_Plac) showed a significant increase in serum ferritin levels between baseline and mid-point (13.31 ng/mL ± 3.72, *p* = 0.0111). When comparing baseline to end-point, serum ferritin levels in the FS65_Plac group showed a significant (16.98 ng/mL ± 4.19, *p* = 0.0049) increase. Notably, when comparing serum ferritin differences between FS65_Plac and FS65_Curc, mid-point to end-point, there was a similar mean increase; 3.67 ng/mL ± 1.94 and 3.04 ng/mL ± 3.74, respectively (Figure 5A).

In the sub-group with serum ferritin ≥ 30 ng/mL, there were no significant changes in mean serum ferritin levels in response to supplementation. There was a non-significant trend for increased serum ferritin when comparing baseline to end-point mean differences, with similar levels recorded for the FS65_Curc (9.38 ng/mL ± 5.82), FS65_Plac (9.46 ng/mL ± 5.73), and the FS18_Curc (9.71 ng/mL ± 5.52) (Table S1).

When sub-grouping according to <50 ng/mL or ≥50 ng/mL, the low serum ferritin sub-group in the FS18_Curc treatment showed a significant increase of 6.20 ng/mL ± 2.38 in mean serum ferritin levels at the mid-point compared to the baseline (*p* = 0.0432) and of 11.00 ng/mL ± 3.94 at end-point compared to baseline (*p* = 0.0352) (Figure 5B). An increase of 7.10 ng/mL ± 1.46 between baseline and mid-point and of 15.00 ng/mL ± 3.77 between baseline and end-point in serum ferritin levels was also observed in the FS65_Curc group, at the significance of *p* = 0.0009 and *p* = 0.0048, respectively (Figure 5B). Furthermore, there were significant serum ferritin increases from baseline for the FS18_Plac group at mid-point (9.30 ng/mL ± 2.84) and end-point (8.00 ng/mL ± 1.98) (*p* = 0.0155 and *p* = 0.0042, respectively) and for the FS65_Plac group at mid-point (11.00 ng/mL ± 2.88) and end-point (17.00 ng/mL ± 3.13) (*p* = 0.0031 and *p* < 0.0001, respectively) (Figure 5B). In the serum ferritin sub-group with ≥50 µg/L ferritin at baseline, there was no significant change in serum ferritin levels (inter- or intra-group comparisons) in relation to supplementation groups.

#### 3.4.2. Effect of Supplementation on Serum Fe Levels

There were no statistically significant changes observed in serum Fe, intra- or inter-group comparisons, as a result of iron and curcumin administration over the duration of 42 days, for any of the treatment groups.

In addition, when participants were sub-grouped according to low baseline serum ferritin (<30 ng/mL or <50 ng/mL) and normal baseline serum ferritin levels (≥30 ng/mL or ≥50 ng/mL), there were no significant changes in serum Fe levels (inter or intra) in the five supplementation groups. 

#### 3.4.3. Effect of Supplementation on Serum TIBC Levels

Statistical analysis did not show any significant difference at baseline, mid-point, or end-point when comparing between the five supplement groups. 

**Figure 6:**
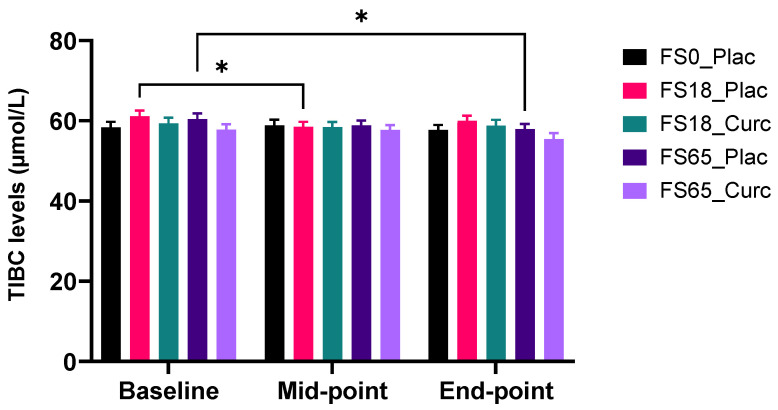
Effect of supplementation on serum TIBC levels. Results are presented as mean ± SEM TIBC levels (µmol/L) in whole group analysis (*n* = 29–31 per group). Serum TIBC was collected and analysed at baseline (day 1), mid-point (day 21), and end-point (day 42). * represents significance values when comparing each condition and time points within the same condition. (* *p* < 0.05).

There is a significant reduction in mean serum TIBC levels within the FS18_Plac group baseline vs. mid-point (2.60 µmol/L ± 0.87, *p* = 0.0152) and the FS65_Plac group baseline vs. end-point (2.45 µmol/L ± 0.82, *p* = 0.0158) (Figure 6).

There were no between-group significant differences observed as a result of iron and curcumin administration over the duration of 42 days.

Sub-group analysis, found that, for those with low serum ferritin (<30 ng/mL), there was a significant serum TIBC reduction observed in the FS65_Plac group from baseline to end-point (4.00 µmol/L ± 1.20) and from mid-point to end-point (2.10 µmol/L ± 0.74) with significance of *p* = 0.0206 and *p* = 0.0404, respectively (Table S1). There were no significant differences observed over time, in sub-groups with baseline ferritin levels of ≥30 ng/mL, as a result of supplementation.

Further sub-group analysis (<50 ng/mL or ≥50 ng/mL serum ferritin at baseline) revealed for the low baseline serum ferritin sub-group (<50 ng/mL), a significant TIBC reduction of 3.63 µmol/L ± 0.94 between baseline and end-point (*p* = 0.0032) and of 2.13 µmol/L ± 0.61 between mid-point and end-point (*p* = 0.0077) in the FS65_Plac treatment group (Table S1). There were no significant differences observed in the sub-groups with baseline ferritin levels of ≥50 ng/mL.

#### 3.4.4. Effect of Supplementation on Serum UIBC Levels

There were no statistically significant changes observed in serum UIBC, intra- or inter-group comparisons, as a result of iron and curcumin administration over the duration of 42 days, when analysing whole groups.

Sub-group analysis, using baseline ferritin levels (low: <30 ng/mL or <50 ng/mL and normal: ≥30 ng/mL or ≥50 ng/mL), showed there was a significant reduction observed in the ≥30 ng/mL sub-group for FS0_Plac, from baseline to end-point, of 6.10 µmol/L ± 1.90 (*p* = 0.0178) (Table S1). There were no other significant changes detected in serum UIBC levels within the rest of the sub-groups over the supplementation period.

#### 3.4.5. Effect of Supplementation on Serum TS%

There were no statistically significant changes observed in serum TS%, intra- or inter-group comparisons, as a result of iron and curcumin administration over the duration of 42 days, when analysing whole groups.

Sub-group analysis, using baseline ferritin levels (low: <30 ng/mL or <50 ng/mL and normal: ≥30 ng/mL or ≥50 ng/mL), revealed a significant increase in the ≥30 ng/mL sub-group for FS0_Plac of 8.60 µmol/L ± 3.30 (*p* = 0.0462) between baseline and end-point. There were no other significant changes detected in serum TS% levels within the rest of the sub-groups over the supplementation period.

### 3.5. Subjective Markers

#### 3.5.1. Gastrointestinal Side-Effects: Frequency of Darker and Black Bowel Movements

Planned comparisons (Fischer’s exact test), with Bonferroni correction, were carried out at all time points; baseline, mid-point, and end-point between FS0_Plac and all other treatment groups; as well as between FS18_Plac vs. FS18_Curc and FS65Plac vs. FS65_Curc (Table S2). At baseline, there were no significant associations between supplementation group and darker bowel movements. However, at mid-point, there were significant associations between supplementation group and darker bowel movements. This was only detected between FS0_Plac vs. FS65_Plac (*p* = 0.002, Fisher’s exact test). Planned comparisons revealed no significant associations at end-point. It appears that combined supplementation of curcumin and iron may potentially decrease the number of darker bowel movements. With respect to frequency of black bowel movements, there were no significant associations between supplementation groups at the different time points. 

#### 3.5.2. Effect of Supplementation on Other Symptoms

In case of other symptoms such as feelings of nausea, vomiting, incidence of heart burn, abdominal pain, headache, breathlessness, and diarrhoea, there were no significant associations between supplementation groups, across the different time points; baseline, mid-point, and end-point (*p* > 0.05).

#### 3.5.3. Effect of Supplementation on Subjective Fatigue Measures 

The self-reported FSS was scored using all 9 items, as well as the 7-item alternative (reported to be more sensitive for detecting change in fatigue), suggested by Lerdal and Kottorp [75]. No significant changes were found between the five supplementation groups, across any of the time points (*p* > 0.05). Similarly, the one-point F-VAS, did not detect any differences between the groups.

## 4. Discussion

Oral iron supplementation has been well established as an effective treatment for ID, with FS the supplement of choice due to its wide availability and low cost [15]. However, its long-term use is commonly associated with adverse effects that are a significant limiting factor that hamper the full potential of this approach [14]. The current study was designed to investigate the co-administration of ferrous sulphate with or without a formulated form of the antioxidant curcumin (HydroCurc™) to assess the potential reduce systemic inflammation, gastrointestinal side-effects, and/or reduce fatigue severity. Due to the relatively large sample size for a study of this kind (154 participants), in addition to analysing the full cohort data, we also analysed the data in a number of ways based on previously reported baseline ferritin threshold levels; 30 ng/mL [70,71,72] and 50 ng/mL [72,74]. The objective was to gain a clear understanding of the influence of initial iron status (in the form of baseline ferritin levels) on impact of supplementation.

Analysing the data as a complete cohort, a significant increase in the plasma TBARS values was evident in the FS18-Placebo group between baseline and end point (Figure 4A) following 6 weeks of daily oral ferrous sulphate (18 mg) indicating that at this dose, ferrous sulphate has increased the levels of oxidative stress. No other treatment groups showed any significant changes in TBARS values. Circulating TBARS levels are used as a chemically stable proxy measurement of lipid peroxidation processes, which result in the accumulation of malondialdehyde (MDA), an end-product of peroxidative decomposition of polyenoic fatty acids [76]. Temporal increases in TBARS in the 18 mg iron supplement group may indicate that low-dose iron increases systemic lipid peroxidation, whereas the addition of formulated curcumin (FS18-Curcumin) prevents this increase. Given this observation at low-dose iron, the lack of observable oxidative stress in the high-dose iron (65 mg/day) group is surprising but does not necessarily imply an absence of such effects. The lack of a significant TBARS effect in the high-dose iron study arm (FS65-Placebo) alone may be indicative of activation and recruitment of additional unclarified endogenous protective processes, which counteract the free radical generating effects of high-dose iron to an extent that there is a lack of an observable significant effect. Indeed, a recent systematic review with meta-analysis investigating GI side-effects of oral iron concluded that oral iron dose was not correlated with the severity of GI side-effects reported [15]. Sub-group analysis of the ≥30 ng/mL ferritin for TBARS also revealed a significant increase in the FS18-Placebo group with an additional increase at the mid time point (21 days) with no significant temporal changes in other groups.

Whole cohort analysis also revealed decreases in plasma TNF levels in the following three groups, FS18-Curcumin, FS65-Placebo, and FS65-Curcumin, between days 21 and 42 only. TNF is an endogenous pyrogen and pro-inflammatory cytokine, which is primarily released from macrophages [77]. Elevated plasma TNF was also demonstrated to be the first link between inflammation and the development of insulin resistance [78]. Anti-TNF therapies are used for the clinical treatment of various auto-immune inflammatory diseases [79]. In the context of the current study, a reduction in baseline levels of TNF may represent a reduction in background circulating inflammation and also aligns with the whole cohort plasma TBARS data, which demonstrated an increase in systemic lipid peroxidation in the FS18-Placebo group alone. FS18-Placebo is the only iron supplementation group to show no decrease in plasma TNF levels between day 21 and 42 and may be an indicator of iron-induced inflammatory side-effects. 

Sub-group analysis of ≥30 ng/mL baseline ferritin for plasma IL-6 also revealed a significant decrease with time, at both 21 and 42 days, compared to baseline levels. This suggests a reduction in systemic inflammation in the presence of formulated curcumin (FS65-Curcumin) but not in the high iron (65 mg/day) alone (FS65-Placebo). IL-6 is both a pro-inflammatory cytokine (and acute phase protein) primarily released by macrophages and an anti-inflammatory cytokine, released from skeletal muscle in response to exercise in proportion to exercise duration and intensity [80]. Given the lack of an exercise component in the current study, it is likely that the observed decreases in baseline levels of circulating IL-6 levels represent a reduction in the pro-inflammatory component primarily released by macrophages and white adipose tissue [81]. Interestingly, IL-6 levels have recently been used to predict severity and survivability in COVID-19 patients as part of a cytokine signature [82], demonstrating the importance of IL-6 as a key early mediator of the inflammatory cascade.

C-reactive protein (CRP) is an acute phase protein synthesised in the liver and increases in response to microbial infection and/or inflammation [83]. Levels of CRP are associated with increasing body mass index (BMI) and body fat, which is linked to obesity-induced low-grade inflammation [84]. In the current study, participants with a BMI in the normal range (≤25) were recruited to avoid any obesity-induced increases in basal CRP levels, which may have added a confounding element to the study results. CRP is known to be under direct transcriptional control from circulating IL-6 [83] as part of the inflammatory cascade of the innate immune system. CRP was not significantly altered by any treatment in the current study despite reductions in circulating IL-6 levels. This may be due to a lack of sensitivity of CRP to falling systemic levels of IL-6 in this healthy population.

The biological effects of IL-10 are generally described as being anti-inflammatory. IL-10 is secreted from specialised Regulatory T cells (T_R_1) and directly reduces the release of pro-inflammatory cytokines such as IL-2 and IL-5 as well as TNF-α and is thought to act as a counter-regulatory factor to add balance to pro-inflammatory activation [85]. It is therefore not surprising perhaps, that with only decreases in pro-inflammatory cytokines recorded in the current study, we saw no significant changes in IL-10 levels with any treatment or time point. 

As may be expected, participants with the lowest baseline plasma ferritin (<30 ng/mL) levels demonstrated the greatest increases in ferritin levels at both days 21 and 42 (see Figure 5A), although only the FS65-Placebo arm was significantly elevated by day 42. Longer lasting effects were seen in the sub-group analysis involving participants in the <50 ng/mL ferritin baseline group, with FS18-Curcumin, FS65-Placebo, and FS65-Curcumin groups all demonstrating a significant increase in day 42 plasma ferritin levels compared to baseline (see Figure 5B). Interestingly, no treatment group or ferritin sub-group analysis demonstrating a significant increase in plasma ferritin levels showed an associated decrease in inflammation-related markers. This observation may be due to the relationship between iron homeostasis and inflammation induced by microbial infection, which results in significant increases in plasma ferritin levels in an attempt to sequester iron from the circulation to actively inhibit bacterial replication in order to mitigate the spread of infection [86]. Furthermore, this suggests that the reduction in circulating inflammation-related markers was predominantly in individuals with adequate iron levels.

The darkened or black stools often associated with long-term oral iron supplementation studies showed a significant association, when it came to dark stool frequency, between placebo control (FS0-Placebo) and the high iron group (FS65-Placebo). This suggests that the addition of curcumin may be protective against darker stools. No significant associations were reported for frequency of black stools. The inclusion of the ‘dark stool’ question to Pereira and colleagues [66] original questionnaire seems to have the ability to detect subtle faecal changes associated with iron supplementation.

We recently demonstrated that HydroCurc™ does not adversely affect oral ferrous sulphate absorption in a human RCT [43], therefore, any difference in systemic biological responses/biomarkers in the current study are unlikely to be caused by the availability or levels of serum iron between treatment groups. Taken together, the results suggest that the presence of curcumin in combination with iron leads to the majority of the observed decreases in inflammation-related markers, compared to iron alone. Furthermore, the only significant increases in serum TBARS (an indicator of free radical activity) were in the low iron group in the absence of curcumin (See Figure 4). No significant increases in TBARS were observed in the presence of curcumin in any of our analyses, suggesting that there may a protective effect of curcumin despite the presence of iron. The results as a whole suggest that curcumin exerts both a protective antioxidative effect by preventing a rise in TBARS and at the same time, and perhaps via different mechanisms, is able to lower selective inflammation-related biomarkers over time. 

Adherence to oral iron treatment is even lower in patients with inflammatory bowel disorders (IBD) compared to the general population. One study on IBD patients reported that 52% of study participants in the oral iron study arm withdrew or reduced ferrous sulphate dose in a multi-centre trial [87]. Given that specific patient groups are more susceptible to serious side-effects of oral iron intake, the current strategy employing oral iron co-administered with a highly absorbed antioxidant formulation may offer a cost effective and more convenient alternative to intra-venous iron infusion for IBD patients or other patient groups at risk of ID. This would require further testing in a suitable clinical population. 

The limitations of the study include the age and baseline iron status of the participants. In this study, participants were young and healthy with normal iron status, i.e., plasma ferritin levels > 15 ng/mL. However, despite this, we were able to measure significant decreases in circulating plasma inflammation-related biomarkers over time, suggesting that there may be wider benefits to populations other than those with ID. The lack of a curcumin-only study arm is a limitation, as we cannot determine whether the reductions in inflammation-related markers observed with curcumin in addition to iron would be more pronounced with curcumin alone. However, the primary focus of the study was to investigate the effects of curcumin on the side-effects of iron supplementation rather than the effects of curcumin on inflammatory status and therefore this treatment group was outside the scope of the current study. We also did not monitor or investigate participant physical activity levels or dietary changes during the study, which should be considered in the future.

## 5. Conclusions

This study indicates that co-administering a formulated, bioavailable form of curcumin alongside ferrous sulphate may reduce systemic inflammation and oxidative stress compared to the same dose of iron alone. Formulated curcumin may thus facilitate the beneficial effects of FS supplementation whilst mitigating some of the undesirable effects that limit the utility of this approach. The results of this study therefore have profound implications, as they provide a direction towards employing newer approaches to oral iron supplementation. Future research exploring the potential of co-formulated FS and curcumin supplements tested in a larger iron deficient cohort would further validate the benefits of this strategy.

## Figures and Tables

**Table 1:** Participant characteristics presented as means ± standard error of mean (SEM).

	Age (y)		Height (m)		Weight (kg)		BMI (kg/m^2^)		Body Fat (%)	
	Male	Female	Male	Female	Male	Female	Male	Female	Male	Female
**FS0_Plac**	25.87 ± 1.18	26.73 ± 1.31	1.77 ± 0.02	1.67 ± 0.02	75.22 ± 3.15	66.36 ± 3.25	24.06 ± 0.83	23.72 ± 0.85	18.37 ± 1.68	32.16 ± 1.57
	(*n* = 31)		(*n* = 30)		(*n* = 30)		(*n* = 30)		(*n* = 30)	
**FS18_Plac**	26.11 ± 1.27	25.08 ± 1.47	1.76 ± 0.01	1.64 ± 0.01	80.70 ± 3.70	60.08 ± 3.07	25.94 ± 1.06	22.38 ± 1.21	21.97 ± 2.02	29.45 ± 2.44
	(*n* = 31)		(*n* = 30)		(*n* = 30)		(*n* = 30)		(*n* = 30)	
**FS18_Curc**	25.60 ± 1.13	23.53 ± 1.24	1.77 ± 0.02	1.59 ± 0.02	74.58 ± 2.78	57.76 ± 2.64	23.71 ± 0.79	22.93 ± 1.03	18.35 ± 1.86	30.29 ± 2.07
	(*n* = 31)		(*n* = 30)		(*n* = 30)		(*n* = 30)		(*n* = 30)	
**FS65_Plac**	26.92 ± 1.25	27.06 ± 1.14	1.78 ± 0.01	1.63 ± 0.02	77.13 ± 2.27	60.27 ± 2.89	24.32 ± 0.56	22.46 ± 0.82	19.13 ± 1.51	27.95 ± 1.52
	(*n* = 30)		(*n* = 30)		(*n* = 30)		(*n* = 30)		(*n* = 30)	
**FS65_Curc**	27.12 ± 1.35	26.36 ± 1.08	1.78 ± 0.02	1.65 ± 0.02	75.24 ± 2.26	58.54 ± 1.91	23.95 ± 0.77	21.48 ± 0.65	19.44 ± 1.85	28.18 ± 1.25
	(*n* = 31)		(*n* = 31)		(*n* = 31)		(*n* = 30)		(*n* = 31)	
**Total**	26.32 ± 0.55	25.81 ± 0.56	1.77 ± 0.01	1.64 ± 0.01	76.69 ± 1.32	60.64 ± 1.28	24.44 ± 0.38	22.61 ± 0.41	19.56 ± 0.82	29.59 ± 0.79
	(*n* = 154)		(*n* = 151)		(*n* = 151)		(*n* = 151)		(*n* = 151)	

## Data Availability

The data presented in this study are available on request from the corresponding author. The data are not publicly available due to ethical, legal, and privacy issues.

## Supplementary Materials

The following supporting information can be downloaded at: https://www.mdpi.com/article/10.3390/nu14030712/s1, Table S1: Comprehensive study results for all ferritin cut-off points and whole cohort analysis for all quantitative markers. Table S2: Subjective markers—darker bowel movement data. Figure S1: The study compliance after 155 participants were enrolled and randomised equally into 5 treatment groups.
