# Supplementary material for: Co-Administration of Iron and Bioavailable Curcumin Reduces Levels of Systemic Markers of Inflammation and Oxidative Stress in a Placebo-Controlled Randomised Study
Source: Nutrients. 2022 Feb 8;14(3):712. doi: 10.3390/nu14030712 (PMC8838381; doi:10.3390/nu14030712)
Supplement: Supplementary file 1 [file nutrients-14-00712-s001.zip › Nutrients-1532209-Supplementary Table S1.pdf]

Supplementary Table S1: Summary results of chronic iron and curcumin supplementation on inflammatory and iron status markers. Results are presented as mean difference, standard error of difference, *p* value and significance where * represents significance values when comparing time points within the same conditions (* $p < 0.05$, ** $p < 0.01$, *** $p < 0.001$, **** $p < 0.0001$); [ns] indicates no significant difference ($p > 0.05$).

Biomarker	Groups	Time points	Whole group						Subgroup analysis based on baseline serum ferritin levels (ng/mL)																							
									<30						≥30						<50						≥50					
			N1	N2	Mean Diff	SE of diff.	*p* value	Sig	N1	N2	Mean Diff	SE of diff.	*p* value	Sig	N1	N2	Mean Diff	SE of diff.	*p* value	Sig	N1	N2	Mean Diff	SE of diff.	*p* value	Sig	N1	N2	Mean Diff	SE of diff.	*p* value	Sig
Plasma CRP (pg/mL)	FS0_Plac	B vs M	30	30	-0.21	0.22	0.5940	ns	13	12	-0.34	0.15	0.0974	ns	17	18	-0.11	0.36	0.9538	ns	17	16	-0.43	0.17	0.0550	ns	13	14	0.07	0.44	0.9877	ns
		B vs E	30	29	-1.08	1.01	0.5383	ns	13	12	-0.31	0.21	0.3490	ns	17	17	-1.61	1.76	0.6380	ns	17	15	-0.22	0.17	0.4365	ns	13	14	-1.95	2.19	0.6549	ns
		M vs E	30	29	-0.87	1.06	0.6959	ns	12	12	0.03	0.25	0.9921	ns	18	17	-1.50	1.80	0.6861	ns	16	15	0.21	0.25	0.6798	ns	14	14	-2.02	2.20	0.6385	ns
	FS18_Plac	B vs M	31	30	-0.21	0.45	0.8902	ns	9	8	-1.61	1.45	0.5400	ns	22	22	0.31	0.29	0.5540	ns	15	14	-0.98	0.84	0.4910	ns	16	16	0.48	0.39	0.4507	ns
		B vs E	31	29	0.06	0.25	0.9721	ns	9	7	-0.44	0.44	0.6135	ns	22	22	0.23	0.30	0.7240	ns	15	13	-0.28	0.26	0.5497	ns	16	16	0.36	0.40	0.6470	ns
		M vs E	30	29	0.27	0.23	0.4806	ns	8	7	1.17	0.80	0.3752	ns	22	22	-0.08	0.24	0.9468	ns	14	13	0.70	0.38	0.2059	ns	16	16	-0.12	0.32	0.9290	ns
	FS18_Curc	B vs M	31	31	0.32	0.35	0.6440	ns	10	10	0.63	0.69	0.6416	ns	21	21	0.17	0.41	0.9137	ns	21	21	0.58	0.50	0.4859	ns	10	10	-0.24	0.25	0.6203	ns
		B vs E	31	31	0.15	0.50	0.9510	ns	10	10	0.62	0.73	0.6778	ns	21	21	-0.08	0.65	0.9926	ns	21	21	0.71	0.55	0.4167	ns	10	10	-1.03	0.95	0.5491	ns
		M vs E	31	31	-0.17	0.28	0.8266	ns	10	10	-0.01	0.19	0.9989	ns	21	21	-0.24	0.41	0.8271	ns	21	21	0.13	0.23	0.8489	ns	10	10	-0.78	0.72	0.5458	ns
	FS65_Plac	B vs M	30	30	0.31	0.28	0.5168	ns	12	12	0.18	0.28	0.7936	ns	18	18	0.40	0.44	0.6406	ns	19	19	0.18	0.18	0.5814	ns	11	11	0.54	0.72	0.7437	ns
		B vs E	30	30	0.29	0.17	0.2081	ns	12	12	0.20	0.31	0.7942	ns	18	18	0.35	0.19	0.1976	ns	19	19	0.23	0.20	0.5066	ns	11	11	0.39	0.30	0.4198	ns
		M vs E	30	30	-0.02	0.22	0.9949	ns	12	12	0.02	0.26	0.9968	ns	18	18	-0.05	0.33	0.9881	ns	19	19	0.05	0.17	0.9544	ns	11	11	-0.14	0.53	0.9616	ns
	FS65_Curc	B vs M	31	29	-0.01	0.31	0.9988	ns	10	9	0.49	0.39	0.4647	ns	21	20	-0.24	0.41	0.8264	ns	16	14	0.45	0.32	0.3813	ns	15	15	-0.45	0.50	0.6512	ns
		B vs E	31	29	0.25	0.15	0.2611	ns	10	9	0.36	0.44	0.6973	ns	21	20	0.19	0.12	0.2345	ns	16	14	0.20	0.28	0.7652	ns	15	15	0.29	0.15	0.1540	ns
		M vs E	29	29	0.26	0.30	0.6603	ns	9	9	-0.13	0.21	0.8246	ns	20	20	0.43	0.42	0.5635	ns	14	14	-0.25	0.22	0.5237	ns	15	15	0.74	0.52	0.3554	ns
Plasma IL-6 (pg/mL)	FS0_Plac	B vs M	29	30	-0.01	0.01	0.4011	ns	12	12	-0.01	0.02	0.7795	ns	17	18	-0.02	0.01	0.5353	ns	16	16	-0.01	0.01	0.5735	ns	13	14	-0.01	0.02	0.7172	ns
		B vs E	29	29	0.00	0.01	0.9816	ns	12	12	0.00	0.01	0.9816	ns	17	17	0.00	0.01	0.9930	ns	16	15	0.01	0.01	0.6750	ns	13	14	0.00	0.02	0.9804	ns
		M vs E	30	29	0.02	0.01	0.2252	ns	12	12	0.01	0.02	0.6663	ns	18	17	0.02	0.01	0.3504	ns	16	15	0.02	0.01	0.2834	ns	14	14	0.01	0.01	0.7197	ns
	FS18_Plac	B vs M	31	29	-0.02	0.05	0.9276	ns	9	8	-0.16	0.08	0.1742	ns	22	21	0.04	0.06	0.8349	ns	15	14	-0.12	0.05	0.0736	ns	16	15	0.08	0.09	0.6386	ns
		B vs E	31	29	0.02	0.05	0.8902	ns	9	7	-0.06	0.03	0.2464	ns	22	22	0.05	0.06	0.6596	ns	15	13	-0.05	0.02	0.0662	ns	16	16	0.09	0.08	0.5281	ns
		M vs E	29	29	0.04	0.02	0.1527	ns	8	7	0.11	0.05	0.1363	ns	21	22	0.01	0.03	0.8290	ns	14	13	0.07	0.04	0.1331	ns	15	16	0.01	0.03	0.9416	ns
	FS18_Curc	B vs M	31	31	-0.01	0.02	0.9025	ns	10	10	0.03	0.04	0.6913	ns	21	21	-0.03	0.02	0.3273	ns	21	21	0.01	0.02	0.9723	ns	10	10	-0.04	0.04	0.5868	ns
		B vs E	31	31	-0.02	0.03	0.7565	ns	10	10	-0.05	0.10	0.8744	ns	21	21	-0.01	0.02	0.7746	ns	21	21	-0.04	0.05	0.7427	ns	10	10	0.00	0.02	0.9963	ns
		M vs E	31	31	-0.02	0.03	0.8548	ns	10	10	-0.08	0.08	0.5580	ns	21	21	0.02	0.02	0.6964	ns	21	21	-0.04	0.04	0.5853	ns	10	10	0.04	0.03	0.3404	ns
	FS65_Plac	B vs M	30	30	-0.01	0.02	0.7587	ns	12	12	-0.03	0.02	0.3270	ns	18	18	0.00	0.02	0.9997	ns	19	19	-0.02	0.01	0.2117	ns	11	11	0.01	0.04	0.9749	ns
		B vs E	30	30	0.00	0.01	0.9859	ns	12	12	-0.02	0.02	0.7272	ns	18	18	0.02	0.02	0.6162	ns	19	19	-0.02	0.02	0.5951	ns	11	11	0.03	0.02	0.3885	ns
		M vs E	30	30	0.01	0.01	0.5876	ns	12	12	0.01	0.03	0.9014	ns	18	18	0.01	0.02	0.5850	ns	19	19	0.01	0.02	0.9053	ns	11	11	0.02	0.03	0.5230	ns
	FS65_Curc	B vs M	31	29	0.02	0.04	0.8650	ns	10	9	0.09	0.12	0.7215	ns	21	20	-0.01	0.03	0.8743	ns	16	14	0.04	0.08	0.8854	ns	15	15	0.00	0.03	0.9915	ns
		B vs E	31	29	0.06	0.04	0.3164	ns	10	9	0.09	0.13	0.7649	ns	21	20	0.04	0.02	0.0479	*	16	14	0.07	0.08	0.6488	ns	15	15	0.05	0.02	0.1484	ns
		M vs E	29	29	0.04	0.02	0.0566	ns	9	9	0.00	0.03	0.9920	ns	20	20	0.06	0.02	0.0073	**	14	14	0.03	0.03	0.4968	ns	15	15	0.04	0.01	0.0255	*
Plasma TNF (pg/mL)	FS0_Plac	B vs M	29	30	-0.44	0.29	0.3089	ns	12	12	-0.73	0.61	0.4835	ns	17	18	-0.23	0.29	0.7041	ns	16	16	-0.64	0.45	0.3586	ns	13	14	-0.20	0.37	0.8579	ns
		B vs E	29	29	0.04	0.16	0.9685	ns	12	12	-0.20	0.16	0.4602	ns	17	17	0.21	0.25	0.6820	ns	16	15	-0.09	0.17	0.8683	ns	13	14	0.19	0.29	0.7854	ns
		M vs E	30	29	0.48	0.29	0.2358	ns	12	12	0.52	0.67	0.7225	ns	18	17	0.44	0.17	0.0510	ns	16	15	0.55	0.53	0.5598	ns	14	14	0.39	0.20	0.1562	ns
	FS18_Plac	B vs M	31	29	-0.15	0.15	0.5862	ns	9	8	-0.56	0.30	0.2116	ns	22	21	0.02	0.17	0.9953	ns	15	14	-0.43	0.18	0.0861	ns	16	15	0.11	0.23	0.8807	ns
		B vs E	31	29	0.11	0.23	0.8679	ns	9	7	-0.33	0.46	0.7608	ns	22	22	0.30	0.26	0.4865	ns	15	13	-0.04	0.29	0.9889	ns	16	16	0.29	0.34	0.6676	ns
		M vs E	29	29	0.27	0.20	0.3805	ns	8	7	0.23	0.18	0.4688	ns	21	22	0.28	0.26	0.5320	ns	14	13	0.39	0.18	0.1173	ns	15	16	0.19	0.34	0.8514	ns
	FS18_Curc	B vs M	31	31	-0.08	0.19	0.9047	ns	10	10	0.03	0.45	0.9979	ns	21	21	-0.13	0.19	0.7662	ns	21	21	-0.12	0.23	0.8603	ns	10	10	0.00	0.35	>0.9999	ns
		B vs E	31	31	0.27	0.21	0.4087	ns	10	10	0.23	0.39	0.8372	ns	21	21	0.29	0.25	0.4886	ns	21	21	0.25	0.23	0.5386	ns	10	10	0.30	0.43	0.7703	ns
		M vs E	31	31	0.35	0.13	0.0288	*	10	10	0.20	0.15	0.4319	ns	21	21	0.42	0.18	0.0657	ns	21	21	0.37	0.16	0.0724	ns	10	10	0.30	0.23	0.4299	ns
	FS65_Plac	B vs M	30	30	-0.29	0.19	0.2984	ns	12	12	-0.62	0.30	0.1359	ns	18	18	-0.06	0.24	0.9626	ns	19	19	-0.26	0.23	0.5104	ns	11	11	-0.33	0.34	0.6094	ns
		B vs E	30	30	0.10	0.19	0.8661	ns	12	12	-0.14	0.23	0.8256	ns	18	18	0.26	0.28	0.6375	ns	19	19	0.01	0.19	0.9983	ns	11	11	0.25	0.43	0.8278	ns
		M vs E	30	30	0.39	0.15	0.0363	*	12	12	0.48	0.20	0.0797	ns	18	18	0.32	0.21	0.3044	ns	19	19	0.27	0.15	0.2082	ns	11	11	0.59	0.31	0.1841	ns
	FS65_Curc	B vs M	31	29	-0.19	0.25	0.7266	ns	10	9	-0.23	0.30	0.7381	ns	21	20	-0.15	0.34	0.8999	ns	16	14	-0.32	0.25	0.4209	ns	15	15	-0.01	0.44	0.9996	ns
		B vs E	31	29	0.46	0.20	0.0653	ns	10	9	0.35	0.31	0.5279	ns	21	20	0.54	0.25	0.1038	ns	16	14	0.41	0.25	0.2718	ns	15	15	0.57	0.31	0.1857	ns
		M vs E	29	29	0.65	0.17	0.0018	**	9	9	0.57	0.20	0.0534	ns	20	20	0.69	0.23	0.0205	*	14	14	0.73	0.19	0.0064	**	15	15	0.59	0.28	0.1291	ns
Plasma IL-1ß (pg/mL)	FS0_Plac	B vs M	29	30	0.00	0.02	0.9949	ns	12	12	-0.02	0.03	0.6703	ns	17	18	0.01	0.02	0.7551	ns	16	16	-0.02	0.02	0.5637	ns	13	14	0.02	0.02	0.6593	ns
		B vs E	29	29	0.01	0.02	0.7975	ns	12	12	-0.02	0.02	0.7157	ns	17	17	0.03	0.02	0.4379	ns	16	15	-0.01	0.01	0.8696	ns	13	14	0.03	0.03	0.5079	ns
		M vs E	30	29	0.01	0.01	0.6500	ns	12	12	0.01	0.03	0.9578	ns	18	17	0.01	0.01	0.1091	ns	16	15	0.01	0.02	0.8473	ns	14	14	0.01	0.01	0.3975	ns
	FS18_Plac	B vs M	31	29	0.04	0.05	0.6964	ns	9	8	0.00	0.01	0.9981	ns	22	21	0.06	0.07	0.6811	ns	15	14	-0.01	0.01	0.7507	ns	16	15	0.09	0.10	0.6643	ns
		B vs E	31	29	0.07	0.06	0.5029	ns	9	7	-0.02	0.02	0.6091	ns	22	22	0.10	0.08	0.4298	ns	15	13	0.00	0.01	0.9642	ns	16	16	0.13	0.11	0.4614	ns
		M vs E	29	29	0.03	0.01	0.1488	ns	8	7	-0.02	0.01	0.4803	ns	21	22	0.04	0.02	0.0538	ns	14	13	0.00	0.01	0.9523	ns	15	16	0.05	0.02	0.1335	ns
	FS18_Curc	B vs M	31	31	0.00	0.01	0.9907	ns	10	10	0.01	0.01	0.8368	ns	21	21	-0.01	0.01	0.8416	ns	21	21	-0.01	0.01	0.8921	ns	10	10	0.01	0.01	0.7342	ns
		B vs E	31	31	0.00	0.01	0.9980	ns	10	10	0.00	0.02	0.9807	ns	21	21	0.00	0.01	0.9961	ns	21	21	0.00	0.01	0.8952	ns	10	10	0.01	0.01	0.6837	ns
		M vs E	31	31	0.00	0.01	0.9942	ns	10	10	-0.01	0.01	0.4843	ns	21	21	0.01	0.01	0.6283	ns	21	21	0.00	0.01	0.9993	ns	10	10	0.00	0.01	0.9759	ns
	FS65_Plac	B vs M	30	30	0.00	0.01	0.9839	ns	12	12	-0.02	0.01	0.0535	ns	18	18	0.01	0.02	0.6803	ns	19	19	0.01	0.01	0.8506	ns	11	11	-0.01	0.01	0.3122	ns
		B vs E	30	30	0.00	0.01	0.8527	ns	12	12	-0.01	0.01	0.4467	ns	18	18	0.01	0.01	0.6775	ns	19	19	0.01	0.01	0.7352	ns	11	11	0.00	0.01	0.6996	ns
		M vs E	30	30	0.00	0.01	0.7903	ns	12	12	0.01	0.01	0.6666	ns	18	18	0.00	0.01	0.9995	ns	19	19	0.00	0.01	0.9121	ns	11	11	0.00	0.01	0.8599	ns
	FS65_Curc	B vs M	31	29	-0.01	0.03	0.8418	ns	10	9	-0.01	0.01	0.5811	ns	21	20	-0.02	0.04	0.9103	ns	16	14	-0.05	0.04	0.4970	ns	15	15	0.02	0.03	0.7524	ns
		B vs E	31	29	0.02	0.01	0.2241	ns	10	9	0.00	0.01	0.9724	ns	21	20	0.04	0.02	0.1792	ns	16	14	0.00	0.01	0.8806	ns	15	15	0.04	0.03	0.2254	ns
		M vs E	29	29	0.04	0.02	0.1747	ns	9	9	0.01	0.01	0.7175	ns	20	20	0.05	0.03	0.2125	ns	14	14	0.05	0.04	0.4455	ns	15	15	0.02	0.01	0.0621	ns
Plasma IL-10 (pg/mL)	FS0_Plac	B vs M	29	30	0.05	0.04	0.4559	ns	12	12	0.02	0.02	0.3664	ns	17	18	0.07	0.07	0.5751	ns	16	16	0.02	0.02	0.2637	ns	13	14	0.09	0.09	0.6303	ns
		B vs E	29	29	0.05	0.05	0.5593	ns	12	12	-0.01	0.04	0.9479	ns	17	17	0.10	0.08	0.4560	ns	16	15	0.00	0.03	0.9916	ns	13	14	0.11	0.10	0.5287	ns
		M vs E	30	29	0.00	0.02	0.9976	ns	12	12	-0.04	0.04	0.6037	ns	18	17	0.02	0.01	0.2720	ns	16	15	-0.02	0.03	0.7746	ns	14	14	0.02	0.02	0.4752	ns
	FS18_Plac	B vs M	31	29	0.05	0.04	0.5168	ns	9	8	0.03	0.06	0.9020	ns	22	21	0.05	0.05	0.5840	ns	15	14	0.03	0.04	0.7208	ns	16	15	0.06	0.07	0.6838	ns
		B vs E	31	29	0.00	0.05	0.9982	ns	9	7	-0.05	0.09	0.8448	ns	22	22	0.02	0.06	0.9362	ns	15	13	-0.04	0.05	0.6844	ns	16	16	0.04	0.08	0.8623	ns
		M vs E	29	29	-0.04	0.03	0.2086	ns	8	7	-0.08	0.06	0.4761	ns	21	22	-0.03	0.03	0.4204	ns	14	13	-0.07	0.04	0.1735	ns	15	16	-0.02	0.03	0.7949	ns
	FS18_Curc	B vs M	31	30	0.03	0.02	0.2806	ns	10	10	0.07	0.05	0.3399	ns	21	20	0.01	0.02	0.7944	ns	21	20	0.03	0.03	0.4808	ns	10	10	0.03	0.03	0.5990	ns
		B vs E	31	31	0.03	0.02	0.4122	ns	10	10	0.04	0.05	0.6509	ns	21	21	0.02	0.02	0.6447	ns	21	21	0.04	0.02	0.2416	ns	10	10	-0.01	0.03	0.9635	ns
		M vs E	30	31	-0.01	0.01	0.8358	ns	10	10	-0.03	0.02	0.2340	ns	20	21	0.00	0.02	0.9729	ns	20	21	0.01	0.02	0.8463	ns	10	10	-0.04	0.02	0.2004	ns
	FS65_Plac	B vs M	30	30	-0.04	0.04	0.5702	ns	12	12	-0.05	0.06	0.6470	ns	18	18	-0.03	0.05	0.8343	ns	19	19	-0.02	0.04	0.8309	ns	11	11	-0.07	0.09	0.7009	ns
		B vs E	30	30	-0.01	0.02	0.8963	ns	12	12	-0.02	0.03	0.7413	ns	18	18	0.00	0.02	0.9812	ns	19	19	-0.01	0.02	0.8262	ns	11	11	0.00	0.03	0.9959	ns
		M vs E	30	30	0.03	0.04	0.6896	ns	12	12	0.03	0.06	0.8833	ns	18	18	0.03	0.05	0.7894	ns	19	19	0.01	0.04	0.9691	ns	11	11	0.07	0.08	0.6700	ns
	FS65_Curc	B vs M	30	28	-0.02	0.04	0.8469	ns	10	9	0.01	0.02	0.8408	ns	20	19	-0.03	0.05	0.7849	ns	15	13	0.01	0.02	0.8601	ns	15	15	-0.04	0.06	0.7908	ns
		B vs E	30	28	0.02	0.01	0.3628	ns	10	9	0.02	0.03	0.7202	ns	20	19	0.02	0.01	0.5159	ns	15	13	0.02	0.02	0.7655	ns	15	15	0.02	0.02	0.3766	ns
		M vs E	28	28	0.04	0.04	0.5733	ns	9	9	0.01	0.01	0.8006	ns	19	19	0.05	0.05	0.6208	ns	13	13	0.01	0.01	0.8240	ns	15	15	0.06	0.07	0.6258	ns
Plasma TBARS (µM)	FS0_Plac	B vs M	29	28	-0.01	0.02	0.8002	ns	12	11	-0.06	0.03	0.1865	ns	17	17	0.02	0.03	0.8319	ns	29	28	-0.01	0.02	0.8002	ns	13	13	0.05	0.03	0.2007	ns
		B vs E	29	27	-0.02	0.03	0.7298	ns	12	11	-0.05	0.05	0.6844	ns	17	16	0.00	0.03	0.9927	ns	29	27	-0.02	0.03	0.7298	ns	13	13	-0.01	0.03	0.9724	ns
		M vs E	28	27	-0.01	0.03	0.9584	ns	11	11	0.01	0.04	0.9601	ns	17	16	-0.02	0.03	0.8270	ns	28	27	-0.01	0.03	0.9584	ns	13	13	-0.06	0.03	0.2142	ns
	FS18_Plac	B vs M	30	29	-0.03	0.02	0.2391	ns	8	7	-0.03	0.05	0.7393	ns	22	22	-0.04	0.02	0.3146	ns	30	29	-0.03	0.02	0.2391	ns	16	16	-0.02	0.03	0.7546	ns
		B vs E	30	29	-0.10	0.04	0.0283	*	8	7	0.05	0.05	0.6303	ns	22	22	-0.15	0.05	0.0079	**	30	29	-0.10	0.04	0.0283	*	16	16	-0.16	0.06	0.0475	*
		M vs E	29	29	-0.07	0.04	0.1403	ns	7	7	0.08	0.03	0.0965	ns	22	22	-0.12	0.04	0.0217	*	29	29	-0.07	0.04	0.1403	ns	16	16	-0.14	0.05	0.0243	*
	FS18_Curc	B vs M	29	29	0.01	0.03	0.9805	ns	9	9	-0.01	0.02	0.9274	ns	20	20	0.01	0.04	0.9589	ns	29	29	0.01	0.03	0.9805	ns	10	10	-0.07	0.03	0.1169	ns
		B vs E	29	29	-0.02	0.05	0.9011	ns	9	9	0.00	0.03	0.9908	ns	20	20	-0.03	0.07	0.8901	ns	29	29	-0.02	0.05	0.9011	ns	10	10	-0.16	0.06	0.0676	ns
		M vs E	29	29	-0.03	0.03	0.696	ns	9	9	0.01	0.03	0.9089	ns	20	20	-0.04	0.04	0.6107	ns	29	29	-0.03	0.03	0.6960	ns	10	10	-0.08	0.06	0.4077	ns
	FS65_Plac	B vs M	28	29	-0.03	0.03	0.4306	ns	11	11	-0.04	0.04	0.6631	ns	17	18	-0.03	0.04	0.6401	ns	28	29	-0.03	0.03	0.4306	ns	10	11	-0.04	0.05	0.7552	ns
		B vs E	28	29	-0.05	0.04	0.4033	ns	11	11	0.03	0.05	0.8499	ns	17	18	-0.09	0.05	0.1672	ns	28	29	-0.05	0.04	0.4033	ns	10	11	-0.09	0.08	0.5481	ns
		M vs E	29	29	-0.01	0.03	0.8807	ns	11	11	0.06	0.05	0.4675	ns	18	18	-0.06	0.03	0.0886	ns	29	29	-0.01	0.03	0.8807	ns	11	11	-0.05	0.03	0.3374	ns
	FS65_Curc	B vs M	30	28	-0.04	0.03	0.4023	ns	10	9	-0.09	0.05	0.2206	ns	20	19	-0.01	0.03	0.9342	ns	30	28	-0.04	0.03	0.4023	ns	14	14	0.02	0.04	0.8506	ns
		B vs E	30	28	-0.07	0.03	0.1084	ns	10	9	-0.17	0.08	0.1318	ns	20	19	-0.03	0.03	0.7041	ns	30	28	-0.07	0.03	0.1084	ns	14	14	0.01	0.04	0.9213	ns
		M vs E	28	28	-0.04	0.04	0.6599	ns	9	9	-0.08	0.11	0.7263	ns	19	19	-0.01	0.03	0.9217	ns	28	28	-0.04	0.04	0.6599	ns	14	14	-0.01	0.04	0.9796	ns
Serum Ferritin (ng/mL)	FS0_Plac	B vs M	30	29	-3.62	2.57	0.3504	ns	13	12	-2.82	2.77	0.5822	ns	17	17	-2.32	4.01	0.8340	ns	17	16	-3.10	2.12	0.3259	ns	13	13	-2.00	5.30	0.9246	ns
		B vs E	30	28	-3.81	3.87	0.5925	ns	13	12	-5.54	5.33	0.5697	ns	17	16	-2.05	5.53	0.9272	ns	17	15	-6.30	4.61	0.3823	ns	13	13	3.50	6.30	0.8438	ns
		M vs E	29	28	-0.19	3.90	0.9986	ns	12	12	-2.72	4.41	0.8143	ns	17	16	0.26	6.04	0.9990	ns	16	15	-3.20	4.14	0.7296	ns	13	13	5.50	6.80	0.7094	ns
	FS18_Plac	B vs M	31	30	0.59	4.03	0.9881	ns	9	8	-5.18	1.31	0.0132	*	22	22	5.03	5.42	0.6289	ns	15	16	-9.30	2.84	0.0155	*	16	16	9.10	6.90	0.4044	ns
		B vs E	31	29	-0.30	3.60	0.9963	ns	9	7	-5.67	2.84	0.1941	ns	22	22	6.08	4.48	0.3810	ns	15	15	-8.00	1.98	0.0042	**	16	16	9.00	6.00	0.3246	ns
		M vs E	30	29	-0.89	3.35	0.9619	ns	8	7	-0.49	3.13	0.9867	ns	22	22	1.06	4.35	0.9681	ns	16	15	1.30	2.72	0.8859	ns	16	16	-0.11	5.60	0.9998	ns
	FS18_Curc	B vs M	31	31	-5.31	2.64	0.1263	ns	11	11	-9.49	3.31	0.0407	*	21	21	-3.30	3.45	0.6124	ns	21	21	-6.20	2.38	0.0432	*	10	10	-3.40	6.70	0.8664	ns
		B vs E	31	31	-8.34	3.80	0.0885	ns	11	11	-11.38	6.30	0.2163	ns	21	21	-9.71	5.52	0.2089	ns	21	21	-11.00	3.94	0.0352	*	10	10	-3.50	8.60	0.9113	ns
		M vs E	31	31	-3.03	4.28	0.7602	ns	11	11	-1.90	7.02	0.9607	ns	21	21	-6.42	5.94	0.5361	ns	21	21	-4.40	4.34	0.5725	ns	10	10	-0.09	10.00	>0.9999	ns
	FS65_Plac	B vs M	30	30	-9.61	2.42	0.0012	**	12	12	-13.31	3.72	0.0111	*	18	18	-7.15	3.14	0.0866	ns	19	19	-11.00	2.88	0.0031	**	11	11	-7.00	4.40	0.3009	ns
		B vs E	30	30	-12.47	3.83	0.0078	**	12	12	-16.98	4.19	0.0049	**	18	18	-9.46	5.73	0.2516	ns	19	19	-17.00	3.13	<0.0001	****	11	11	-3.80	8.60	0.8970	ns
		M vs E	30	30	-2.86	2.97	0.6064	ns	12	12	-3.67	1.94	0.1860	ns	18	18	-2.32	4.84	0.8822	ns	19	19	-6.30	2.65	0.0691	ns	11	11	3.10	6.50	0.8822	ns
	FS65_Curc	B vs M	31	29	-6.60	2.92	0.0781	ns	10	9	-5.04	1.70	0.0430	*	21	20	-6.15	4.20	0.3290	ns	16	14	-7.10	1.46	0.0009	***	15	15	-1.30	5.50	0.9686	ns
		B vs E	31	29	-9.77	4.08	0.0589	ns	10	9	-8.08	3.04	0.0668	ns	21	20	-9.38	5.82	0.2648	ns	16	14	-15.00	3.77	0.0048	**	15	15	-0.31	6.70	0.9988	ns
		M vs E	29	29	-3.17	3.11	0.5722	ns	9	9	-3.04	3.74	0.7047	ns	20	20	-3.23	4.25	0.7323	ns	14	14	-7.60	3.62	0.1257	ns	15	15	1.00	4.90	0.9766	ns
Serum Iron (µmol/L)	FS0_Plac	B vs M	31	30	-1.28	1.48	0.8603	ns	13	12	-0.79	2.26	0.9352	ns	18	18	-1.60	2.00	0.7112	ns	17	16	-0.84	1.78	0.8861	ns	14	14	-1.69	2.51	0.7833	ns
		B vs E	31	29	-2.63	1.29	0.6132	ns	13	12	-0.68	2.00	0.9382	ns	18	17	-4.00	1.70	0.0685	ns	17	15	-2.23	1.80	0.4499	ns	14	14	-2.87	1.92	0.3252	ns
		M vs E	30	29	-1.35	1.16	0.1533	ns	12	12	0.11	2.06	0.9985	ns	18	17	-2.40	1.30	0.2056	ns	16	15	-1.40	1.92	0.7529	ns	14	14	-1.19	1.31	0.6451	ns
	FS18_Plac	B vs M	31	30	0.79	1.52	0.8603	ns	9	8	0.24	1.63	0.9880	ns	22	22	1.20	2.00	0.8350	ns	15	14	1.91	1.95	0.6036	ns	16	16	-0.06	2.32	0.9996	ns
		B vs E	31	29	-1.45	1.52	0.6132	ns	9	7	-1.66	1.92	0.6803	ns	22	22	-1.10	2.00	0.8506	ns	15	13	-0.84	1.74	0.8799	ns	16	16	-1.71	2.45	0.7696	ns
		M vs E	30	29	-2.24	1.17	0.1533	ns	8	7	-1.90	2.18	0.6754	ns	22	22	-2.20	1.40	0.2784	ns	14	13	-2.75	1.70	0.2760	ns	16	16	-1.64	1.66	0.5943	ns
	FS18_Curc	B vs M	31	31	0.93	1.45	0.7982	ns	10	10	0.52	2.38	0.9741	ns	21	21	1.10	1.90	0.8176	ns	21	21	1.03	1.91	0.8522	ns	10	10	0.72	2.18	0.9419	ns
		B vs E	31	31	0.27	1.50	0.9819	ns	10	10	0.46	2.73	0.9845	ns	21	21	0.19	1.80	0.9944	ns	21	21	0.56	1.71	0.9433	ns	10	10	-0.32	3.11	0.9942	ns
		M vs E	31	31	-0.66	1.26	0.8618	ns	10	10	-0.06	1.55	0.9992	ns	21	21	-0.94	1.70	0.8508	ns	21	21	-0.48	1.35	0.9338	ns	10	10	-1.04	2.83	0.9286	ns
	FS65_Plac	B vs M	30	30	-1.26	1.99	0.8034	ns	12	12	-1.77	3.16	0.8439	ns	18	18	-0.92	2.60	0.9351	ns	19	19	-1.67	2.66	0.8070	ns	11	11	-0.55	3.05	0.9820	ns
		B vs E	30	30	1.29	1.65	0.7191	ns	12	12	1.07	2.15	0.8750	ns	18	18	1.40	2.40	0.8236	ns	19	19	1.66	2.07	0.7055	ns	11	11	0.64	2.87	0.9734	ns
		M vs E	30	30	2.55	2.34	0.5296	ns	12	12	2.83	3.58	0.7162	ns	18	18	2.40	3.20	0.7420	ns	19	19	3.33	2.82	0.4795	ns	11	11	1.19	4.28	0.9585	ns
	FS65_Curc	B vs M	30	29	-1.37	2.16	0.8026	ns	10	9	-6.20	5.45	0.5199	ns	20	20	0.98	1.80	0.8463	ns	16	14	-3.02	3.95	0.7305	ns	14	15	0.18	1.85	0.9949	ns
		B vs E	30	29	-0.63	2.19	0.9557	ns	10	9	-3.60	4.89	0.7499	ns	20	20	0.90	2.30	0.9187	ns	16	14	0.68	3.80	0.9826	ns	14	15	-1.83	2.23	0.6966	ns
		M vs E	29	29	0.74	1.80	0.9101	ns	9	9	2.60	4.00	0.7974	ns	20	20	-0.09	1.90	0.9988	ns	14	14	3.70	2.85	0.4214	ns	15	15	-2.01	2.08	0.6084	ns
Serum TIBC (µmol/L)	FS0_Plac	B vs M	31	30	-0.55	0.74	0.7454	ns	13	12	-1.60	1.10	0.3457	ns	18	18	0.00	1.00	>0.9999	ns	17	16	-1.45	1.00	0.3471	ns	14	14	0.29	1.11	0.9643	ns
		B vs E	31	29	0.67	0.85	0.7186	ns	13	12	-0.35	1.40	0.9679	ns	18	17	1.30	1.10	0.4583	ns	17	15	-0.08	1.33	0.9979	ns	14	14	1.07	1.08	0.5954	ns
		M vs E	30	29	1.21	0.67	0.1848	ns	12	12	1.30	0.58	0.1230	ns	18	17	1.30	1.10	0.4514	ns	16	15	1.36	0.89	0.3092	ns	14	14	0.79	1.03	0.7329	ns
	FS18_Plac	B vs M	31	30	2.60	0.87	0.0152	*	9	8	3.20	1.50	0.1354	ns	22	22	2.20	1.10	0.1201	ns	15	14	3.53	1.54	0.0924	ns	16	16	1.69	0.91	0.1844	ns
		B vs E	31	29	1.06	0.81	0.3981	ns	9	7	1.10	1.90	0.8329	ns	22	22	0.77	0.90	0.6713	ns	15	13	0.14	1.30	0.9938	ns	16	16	1.63	1.01	0.2736	ns
		M vs E	30	29	-1.53	1.09	0.3499	ns	8	7	-2.10	0.99	0.1588	ns	22	22	-1.50	1.40	0.5701	ns	14	13	-3.39	1.95	0.2308	ns	16	16	-0.06	1.07	0.9981	ns
	FS18_Curc	B vs M	31	31	0.94	0.72	0.4064	ns	10	10	1.80	1.30	0.3647	ns	21	21	0.52	0.89	0.8261	ns	21	21	1.33	0.88	0.3047	ns	10	10	0.10	1.28	0.9966	ns
		B vs E	31	31	0.61	1.04	0.8261	ns	10	10	4.00	1.70	0.0959	ns	21	21	-1.00	1.20	0.6747	ns	21	21	1.52	1.30	0.4816	ns	10	10	-1.30	1.63	0.7147	ns
		M vs E	31	31	-0.32	0.74	0.8997	ns	10	10	2.20	1.00	0.1375	ns	21	21	-1.50	0.86	0.2069	ns	21	21	0.19	0.90	0.9756	ns	10	10	-1.40	1.28	0.5390	ns
	FS65_Plac	B vs M	29	29	1.52	0.71	0.1015	ns	12	11	1.90	1.20	0.3007	ns	17	18	1.20	0.90	0.3894	ns	19	18	1.51	0.86	0.2133	ns	10	11	1.41	1.32	0.5540	ns
		B vs E	29	30	2.45	0.82	0.0158	*	12	12	4.00	1.20	0.0206	*	17	18	1.40	1.00	0.4049	ns	19	19	3.63	0.94	0.0032	**	10	11	0.32	1.37	0.9706	ns
		M vs E	29	30	0.93	0.62	0.3018	ns	11	12	2.10	0.74	0.0404	*	18	18	0.17	0.87	0.9801	ns	18	19	2.13	0.61	0.0077	**	11	11	-1.09	1.10	0.5978	ns
	FS65_Curc	B vs M	30	29	0.07	1.08	0.9974	ns	10	9	0.82	1.80	0.8966	ns	20	20	-0.40	1.40	0.9530	ns	16	14	0.96	1.22	0.7178	ns	14	15	-0.95	1.80	0.8592	ns
		B vs E	30	29	2.43	1.50	0.2542	ns	10	9	6.50	4.10	0.3046	ns	20	20	0.45	0.85	0.8580	ns	16	14	4.56	2.79	0.2677	ns	14	15	0.25	0.97	0.9650	ns
		M vs E	29	29	2.36	1.64	0.3363	ns	9	9	5.70	4.30	0.4233	ns	20	20	0.85	1.40	0.8068	ns	14	14	3.60	2.89	0.4497	ns	15	15	1.20	1.72	0.7691	ns
Serum UIBC (µmol/L)	FS0_Plac	B vs M	30	29	1.07	1.78	0.8194	ns	13	12	-0.78	2.60	0.9529	ns	17	17	2.20	2.40	0.6328	ns	17	16	-0.62	2.15	0.9551	ns	14	14	2.14	2.86	0.7388	ns
		B vs E	30	28	3.64	1.67	0.0919	ns	13	12	0.34	2.80	0.9916	ns	17	16	6.10	1.90	0.0178	*	17	15	2.07	2.31	0.6509	ns	14	14	4.02	2.39	0.2480	ns
		M vs E	29	28	2.57	1.27	0.1265	ns	12	12	1.10	2.10	0.8517	ns	17	16	3.80	1.60	0.0753	ns	16	15	2.69	1.99	0.3899	ns	14	14	1.88	1.45	0.4236	ns
	FS18_Plac	B vs M	31	30	1.79	1.55	0.4889	ns	9	8	2.90	2.70	0.5551	ns	22	22	1.10	1.90	0.8373	ns	15	14	1.47	2.27	0.7965	ns	16	16	1.86	2.19	0.6793	ns
		B vs E	31	29	2.60	1.74	0.3088	ns	9	7	2.90	2.20	0.4549	ns	22	22	1.90	2.20	0.6656	ns	15	13	0.98	1.88	0.8609	ns	16	16	3.51	2.83	0.4505	ns
		M vs E	30	29	0.81	1.31	0.8109	ns	8	7	-0.09	2.00	0.9990	ns	22	22	0.86	1.60	0.8587	ns	14	13	-0.49	1.84	0.9623	ns	16	16	1.64	1.85	0.6567	ns
	FS18_Curc	B vs M	31	31	0.06	1.61	0.9993	ns	10	10	1.30	2.90	0.8988	ns	21	21	-0.52	2.00	0.9620	ns	21	21	0.29	2.11	0.9900	ns	10	10	-0.42	2.44	0.9838	ns
		B vs E	31	31	0.38	1.57	0.9682	ns	10	10	3.50	3.40	0.5786	ns	21	21	-1.10	1.60	0.7863	ns	21	21	1.02	2.01	0.8682	ns	10	10	-0.97	2.51	0.9214	ns
		M vs E	31	31	0.32	1.44	0.9728	ns	10	10	2.20	1.90	0.5018	ns	21	21	-0.57	1.90	0.9537	ns	21	21	0.74	1.63	0.8937	ns	10	10	-0.55	3.00	0.9816	ns
	FS65_Plac	B vs M	29	29	1.16	1.83	0.8028	ns	12	11	0.31	2.70	0.9928	ns	17	18	1.40	2.50	0.8464	ns	19	18	1.10	2.50	0.8992	ns	10	11	0.98	2.66	0.9284	ns
		B vs E	29	30	0.96	1.94	0.874	ns	11	12	3.00	2.60	0.5096	ns	17	18	-0.53	2.70	0.9792	ns	19	18	1.98	2.46	0.7031	ns	10	11	-0.99	3.20	0.9486	ns
		M vs E	29	30	-0.20	1.96	0.9942	ns	11	12	2.70	1.50	0.2235	ns	18	18	-1.90	3.00	0.7990	ns	18	19	0.88	2.08	0.9054	ns	11	11	-1.97	4.00	0.8759	ns
	FS65_Curc	B vs M	30	29	1.51	2.24	0.7806	ns	10	9	7.00	4.80	0.3580	ns	20	20	-1.30	2.20	0.8305	ns	16	14	4.00	3.65	0.5332	ns	14	15	-1.04	2.54	0.9131	ns
		B vs E	30	29	0.58	1.65	0.935	ns	10	9	1.80	1.80	0.6103	ns	20	20	-0.28	2.30	0.9915	ns	16	14	-1.41	2.26	0.8101	ns	14	15	2.22	2.36	0.6252	ns
		M vs E	29	29	-0.93	2.16	0.9038	ns	9	9	-5.20	4.30	0.4857	ns	20	20	0.99	2.40	0.9117	ns	14	14	-5.41	2.83	0.1745	ns	15	15	3.25	2.92	0.5218	ns
Serum TS (%)	FS0_Plac	B vs M	30	29	-2.01	2.76	0.7503	ns	13	12	0.10	3.70	0.9996	ns	17	17	-3.40	4.00	0.6754	ns	17	16	0.00	2.89	>0.9999	ns	14	14	-2.71	4.97	0.8502	ns
		B vs E	30	28	-5.37	2.52	0.1031	ns	13	12	-0.99	3.70	0.9624	ns	17	16	-8.60	3.30	0.0462	*	17	15	-3.61	3.23	0.5200	ns	14	14	-5.86	3.86	0.3154	ns
		M vs E	29	28	-3.36	2.01	0.2332	ns	12	12	-1.10	3.30	0.9441	ns	17	16	-5.20	2.50	0.1228	ns	16	15	-3.60	3.13	0.5001	ns	14	14	-3.14	2.35	0.4014	ns
	FS18_Plac	B vs M	31	30	0.20	2.59	0.9966	ns	9	8	-0.88	2.90	0.9503	ns	22	22	0.91	3.40	0.9619	ns	15	14	1.43	3.23	0.8982	ns	16	16	-0.63	4.05	0.9870	ns
		B vs E	31	29	-2.78	2.61	0.5421	ns	9	7	-3.30	3.10	0.5633	ns	22	22	-2.00	3.40	0.8249	ns	15	13	-1.53	2.93	0.8624	ns	16	16	-3.31	4.22	0.7177	ns
		M vs E	30	29	-2.99	1.81	0.2406	ns	8	7	-2.40	3.30	0.7581	ns	22	22	-2.90	2.20	0.3916	ns	14	13	-2.96	2.46	0.4741	ns	16	16	-2.69	2.67	0.5846	ns
	FS18_Curc	B vs M	31	31	2.00	2.64	0.7308	ns	10	10	1.60	4.90	0.9439	ns	21	21	2.20	3.20	0.7738	ns	21	21	2.33	3.51	0.7858	ns	10	10	1.30	3.79	0.9376	ns
		B vs E	31	31	1.00	2.60	0.9217	ns	10	10	0.00	5.50	>0.9999	ns	21	21	1.50	2.90	0.8672	ns	21	21	1.00	3.16	0.9463	ns	10	10	1.00	4.81	0.9766	ns
		M vs E	31	31	-1.00	2.16	0.8889	ns	10	10	-1.60	2.80	0.8368	ns	21	21	-0.71	2.90	0.9680	ns	21	21	-1.33	2.34	0.8377	ns	10	10	-0.30	4.74	0.9978	ns
	FS65_Plac	B vs M	29	29	-0.31	3.10	0.9945	ns	12	11	1.50	3.60	0.9036	ns	17	18	-1.10	4.60	0.9704	ns	19	18	-0.32	4.05	0.9966	ns	10	11	-0.01	5.00	>0.9999	ns
		B vs E	29	30	1.29	3.00	0.9029	ns	12	12	0.00	3.80	>0.9999	ns	17	18	2.30	4.40	0.8629	ns	19	19	0.53	3.92	0.9901	ns	10	11	2.81	4.76	0.8289	ns
		M vs E	29	30	1.61	3.51	0.8919	ns	11	12	-1.50	2.80	0.8529	ns	18	18	3.40	5.50	0.8110	ns	18	19	0.85	3.79	0.9730	ns	11	11	2.82	7.18	0.9192	ns
	FS65_Curc	B vs M	30	29	-2.58	3.46	0.7387	ns	10	9	-9.20	8.10	0.5201	ns	20	20	0.80	3.30	0.9674	ns	16	14	-4.38	6.13	0.7596	ns	14	15	-0.86	3.42	0.9657	ns
		B vs E	30	29	-0.24	3.04	0.9966	ns	10	9	-1.50	3.90	0.9241	ns	20	20	0.70	4.10	0.9844	ns	16	14	4.40	4.40	0.5891	ns	14	15	-4.53	3.90	0.4949	ns
		M vs E	29	29	2.34	3.09	0.7316	ns	9	9	7.80	5.70	0.4062	ns	20	20	-0.10	3.60	0.9996	ns	14	14	8.78	4.14	0.1239	ns	15	15	-3.67	4.09	0.6516	ns
