# Supplementary material for: Co-Administration of Iron and Bioavailable Curcumin Reduces Levels of Systemic Markers of Inflammation and Oxidative Stress in a Placebo-Controlled Randomised Study
Source: Nutrients. 2022 Feb 8;14(3):712. doi: 10.3390/nu14030712 (PMC8838381; doi:10.3390/nu14030712)
Supplement: Supplementary file 1 [file nutrients-14-00712-s001.zip › Nutrients-1532209-Supplementary Table S2& Figure .pdf]

Darker Bowel Movement – Base					Darker Bowel Movement - Mid-point					Darker Bowel Movement - End-point				
FS0_Plac	FS18_Plac	FS18_Curc	FS65_Plac	FS65_Curc	FS0_Plac	FS18_Plac	FS18_Curc	FS65_Plac	FS65_Curc	FS0_Plac	FS18_Plac	FS18_Curc	FS65_Plac	FS65_Curc
X	X	X	X	X	X	X	X	X	X	X	X	X	X	X
p =.547	X	X	X	X	p = .071	X	X	X	X	p = .070	X	X	X	X
p = 1.000	p = .416	X	X	X	p = .610	p = .111	X	X	X	p = 1.000	p = .212	X	X	X
p = .498	X	X	X	X	p = .002	X	X	X	X	p = .015	X	X	X	X
p = 1.000	X	X	p = 1.000	X	p = .032	X	X	p = .472	X	p = .028	X	X	p = .912	X

Table S2. Outcome of planned Comparisons (Fisher's exact test) with Bonferroni correction*, for darker bowel movements among all treatment groups and across all time points.

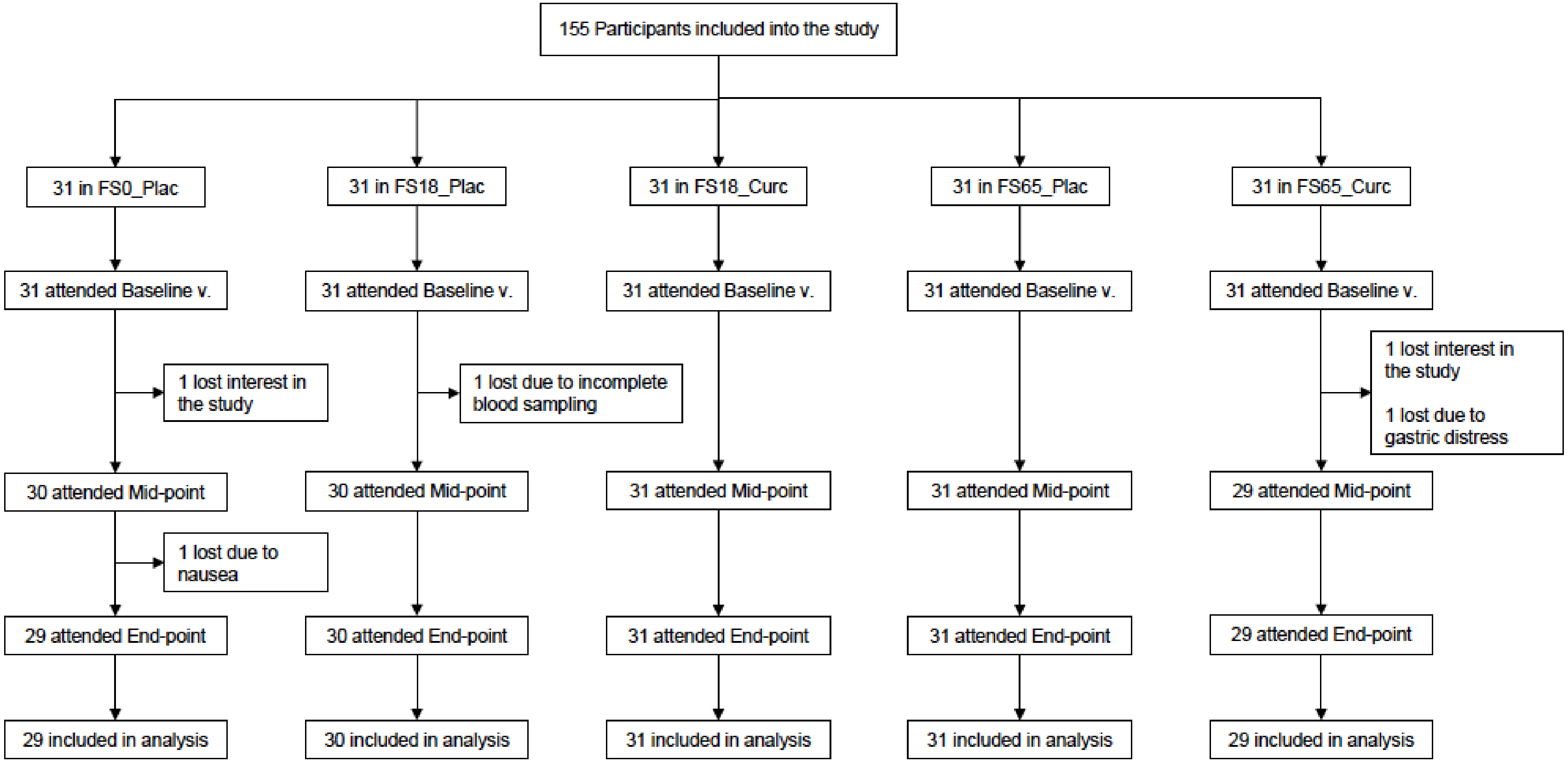

Figure S1. Study compliance after 155 participants were enrolled and randomised equally into 5 treatment groups: FS0_Plac (full placebo, placebos for both iron and curcumin), FS18+Plac (18 mg elemental iron and placebo for curcumin), FS18_Curc (18 mg elemental iron and 500 mg curcumin), FS65_Plac (65 mg elemental iron and placebo for curcumin) and FS65_Curc (65 mg elemental iron and 500 mg curcumin).
